# An ARF1-binding factor triggering programmed cell death and periderm development in pear russet fruit skin

**DOI:** 10.1093/hr/uhab061

**Published:** 2022-01-19

**Authors:** Yuezhi Wang, Meisong Dai, Xinyi Wu, Shujun Zhang, Zebin Shi, Danying Cai, Lixiang Miao

**Affiliations:** 1 Institute of Horticulture, Zhejiang Academy of Agricultural Sciences, Shiqiao Road No. 139, Hangzhou, Zhejiang Province, 310021, China; 2 Institute of Vegetable, Zhejiang Academy of Agricultural Sciences, Desheng Middle Road No. 298, Hangzhou, Zhejiang Province, 310021, China

## Abstract

Plants have a cuticular membrane (CM) and periderm membrane (PM), which act as barriers to terrestrial stresses. The CM covers primary organs with a continuous hydrophobic layer of waxes embedded in cutin, while the PM includes suberized cells stacked externally to the secondary tissues. The formation of native periderm is regulated by a postembryonic meristem phellogen that produces suberized phellem (cork) outwardly. However, the mechanism controlling phellogen differentiation to phellem remains to be clarified. Here, map-based cloning in a pear *F*_**1**_ population with segregation for periderm development in fruit skin facilitated the identification of an aspartic acid repeat deletion in *Pyrus* Periderm Programmed Cell Death 1.1 (PyPPCD1.1) that triggers phellogen activity for cork formation in russet fruit skin of pear. *PyPPCD1.1* showed preferential expression in pear fruit skin, and the encoded protein shares a structural similarity to that of the viral capsid proteins. Aspartic acid deletion in PyPPCD1.1 weakened its nuclear localization but increased its accumulation in the chloroplast. The products of both *PyPPCD1.1* and its recessive allele directly interact with ADP-ribosylation factor 1 (ARF1). PyPPCD1.1 triggered programmed cell death in an ARF1-dependent manner. Thus, this study identified the switch gene for programmed cell death and periderm development and provided a new molecular regulatory mechanism underlying the development of this trait.

## Introduction

Plant species have overcome a series of environmental challenges during their evolution from aquatic to terrestrial habitats. The outermost cork layer stacked on the periderm replaces the cuticular membrane as the barrier against pathogens and reduces water loss in most eudicots and gymnosperms during their secondary growth [1–4]. The biochemical composition of cork includes the main components of suberin and suberin-associated waxes in the suberin complex of the lamellated secondary wall [5–7]. Cork is also enriched in diverse specialized metabolites, such as phenolics, terpenes, alkaloids, and bioactive compounds [8, 9]. Together, these components, combined with the unique structure of the periderm, contribute to the defensive function of cork against environmental stresses. Moreover, the chemical and physical properties of phellem include well-organized suberized cells in some species that render it an optimal raw material for many industrial products, such as sealants, wine stoppers, and surfacing panels [10, 11].

Periderm formation is actively regulated by a bifacial postembryonic meristem phellogen that produces phelloderm inwardly and phellem (cork layer) outwardly [2, 12]. In *Arabidopsis* roots, the development of periderm growth starts with the first cell division at the xylem pole pericycle. Then, the pericycle divides periclinally, the endodermal cells shrink and flatten, the outer tissues loosen with the endodermis undergoing programmed cell death (PCD), and the epidermis and the cortex peel off [13]. Different from that described for root and hypocotyl periderm in *Arabidopsis*, the phellogen in kiwi fruit skin originates in the underlying hypodermal cells that undergo dedifferentiation to re-enter the cell cycle [14]. Periderm development has been investigated in depth, considering its importance in plant performance and survival as well as industrial products. The complex chemical composition of the periderm suggested that its development is involved in multiple secondary metabolic pathways, as revealed by the comprehensive analysis of the transcriptomes and/or proteomes of the tissues differentiated in cork accumulation [7, 15–17]. Due to its protective function, the periderm is also enriched in diverse stress-responsive transcripts, proteins, and networks [15–17]. In addition, phellem differentiation genes have been characterized in *Arabidopsis* and non-model species, and a set of MYB and NAC transcription factor family members showed similar roles in angiosperms with respect to suberin formation processes [7, 13, 18–21].

**Figure 1:**
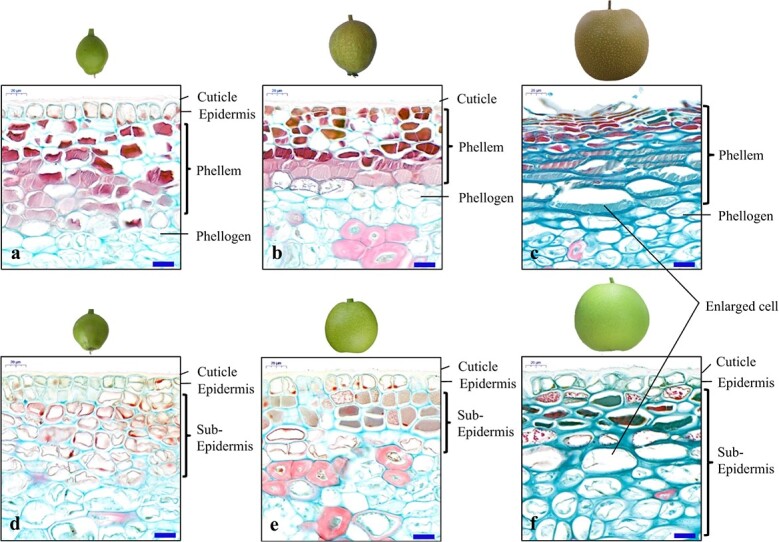
Differentiation in periderm development between russet and green fruit skin of sand pear at 14 DAA (**a**, **d**), 30 DAA (**b**, **e**), and 80 DAA (**c**, **f**). **a**–**c**. The development of periderm in russet fruit skin involves the dedifferentiation of subepidermal parenchymatic cells into phellogen and subsequent cork formation shifting to cell death. The suberized cells die and are compressed in the mature periderm, with the outermost layer of dead cells shrivelling, cracking, and peeling off at 80 DAA (c). **d**–**f** The epidermis is maintained in the green fruit skin at all stages. Bars in microscopic views: 20 μm.

When plants enter secondary growth, the development of the cuticular membrane switches to that of the periderm membrane, which may be regulated by a switch gene. Although the regulators and pathways underlying periderm formation have been revealed, the switch gene controlling phellogen establishment and activity is unclear. The suberized cells undergo PCD during the development of phellem. Typically, PCD causes an actively controlled cellular suicide to fulfil several developmental, differentiation-related, tissue homeostatic, and immune functions [22, 23]. Several plant developmental programs involve a PCD step that is crucial to periderm development [13, 24]; however, the onset of PCD and its role in periderm development have yet to be clarified. As PCD mutations are often fatal, obtaining appropriate genetic materials from model plants for cloning the switch gene that triggers PCD or periderm regulation is challenging. Pear trees (*Pyrus* L., subfamily Pomoideae in the family Rosaceae) have a long history (>3000 years) of cultivation [25]; the fruit is popular for its delicious flavour and high health and economic value. The proportion of the fruit’s area occupied by a cork layer varies and creates a partial russet phenotype in the fruit skin, which affects both the appearance and the shelf life of the fruit and has attracted broad interest among researchers [14, 15, 26]. Compared with green fruit skin, which maintains an epidermis throughout life, the periderm develops on the surface of russet fruits and involves the dedifferentiation of subepidermal parenchymatic cells into the cork cambium and subsequent suberization to cell death to accumulate the cork layer (Fig. 1). Although periderm development in pear fruit skin is regulated by a complex network [17, 27], the sand pear full russet trait of fruit skin shows a monogenic characteristic controlled by a dominant gene [26]. This characteristic of the fruit skin makes sand pear a suitable material for the identification of the gene that triggers phellogen differentiation into cell suberization and death for cork formation in periderm development. The present study first identified *periderm programmed cell death 1.1* (*PyPPCD1.1*), which initiates periderm development in pear fruit skin, through map-based candidate gene screening and functional verification. The analysis of heterologous expression revealed that *PyPPCD1.1* triggers cell death in different plant species. The mechanism underlying PyPPCD1.1*-*guided PCD was uncovered by protein interaction and functional analysis. PyPPCD1.1 binds to ADP-ribosylation factor 1 (ARF1) and triggers ARF1-dependent cell death. Phylogenetic analysis showed the functional conservation of PyPPCD1.1 homologues among woody plants and differentiation between woody and herbaceous species. Due to the involvement of ARF1 in pathogenic invasion, non-host resistance, and R gene-mediated resistance [28–31], this study also revealed that developmental PCD during periderm development shares the core effectors of PCD induced by pathogenic invasion. Furthermore, the periderm-formation-determining gene *PyPPCD1* could be a vital tool in pioneering breeding for plant stress resistance, cork production, and fruit quality.

## Results

### Fine mapping and screening of the switch gene triggering the russeting of pear fruit skin

**Figure 2:**
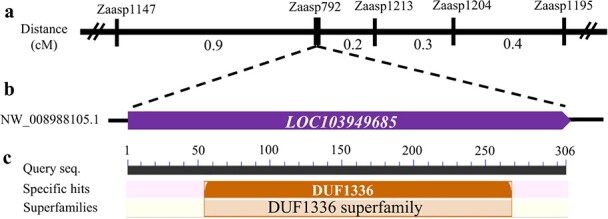
Fine mapping of the gene controlling the russet trait of pear fruit skin. **a** The region on linkage group 8 where the russet locus was mapped. **b** The locus of the only candidate gene in the genome assembly sequence, NW_008988105.1. **c** Homologous annotation of the candidate gene.

Compared with the green sand pear fruit sealed by a cuticular membrane across its entire developmental process, the periderm membrane in the russet sand pear fruit involved continuous suberization and cell death in the phellem and cork formation (Fig. 1). The segregation rates of the russet and non-russet (semi-russet and green) fruit skin in the *F*_1_ progenies were consistent with a ratio of 1:1 from the 12 crosses of the russet fruit skin genotype × non-russet fruit skin genotype (Supplementary Table S1), indicating that the russet trait was inherited independently of the intermediate and green traits and was controlled by a dominant gene*.* High-throughput analysis was employed to fine map the genes responsible for the russet trait of pear fruit skin. Since it is a dominant trait, >30 *F*_1_ progenies each from russet and green skin fruits originating from the cross between sand pear cv. ‘Qingxiang’ (russet fruit skin) and ‘Cuiguan’ (intermediate fruit skin) were pooled for whole-genome sequencing. Consequently, single-nucleotide polymorphisms (SNPs) were detected, and the SNP index values were compared between the two gene pools. The SNP loci conformed to a 1:1 ratio of heterozygous to homozygous reads, as assessed by the χ^2^ test in the russet pool, but showed homozygosity or different heterozygous types in the green pool, which was used for chromosome enrichment analysis. A total of 1770 SNP loci meeting these requirements showed enrichment on linkage group 8, with a peak distribution at the end of the chromosome (Supplementary Fig. S1A, B). The 25 SNPs and 32 polymorphic simple sequence repeats (SSRs) (Supplementary Tables S2 and S3) in the SNP-enriched region were selected for gene linkage analysis in the *F*_1_ population. Finally, the russet gene was located near the cosegregated SSR marker *Zaasp792* with proximal markers within 0.2 and 0.9 cM on either side (Fig. 2; Supplementary Fig. S1C).

Alignment of the primer sequences for *Zaasp1147* and *Zaasp1213* against the reference genome revealed 48 genes with detectable expression in pear fruit skin between these markers. Except for 17 genes with low expression in both russet and green fruit skin samples, the remaining genes showed high transcript levels in both samples (Supplementary Table S4). Thus, the differences in the expression of these genes between the parents could not satisfy the functional characteristics of the switch gene, i.e. no significant difference in the level of expression was detected between the parents. Therefore, alleles of the russet gene may be important for protein function variation rather than gene expression differences. Genome sequencing of both parents was performed to detect non-synonymous mutations among the coding regions of the genes surrounding the russet gene locus. The results revealed a G/T SNP and an SSR polymorphism (a GAC repeat change detected by *Zaasp792*) in the only exon of *LOC103949685*; these polymorphisms resulted in a non-synonymous Gln^249^ → His (Q249H) substitution and an Asp deletion in the C-terminal Asp tandem repeat, respectively, in the russet skin samples. *LOC103949685* alleles in both parents encoded unknown proteins belonging to the DUF1336 superfamily with six (allele A), seven (allele B and allele C), and eight (allele D) Asp tandem repeats at the C-terminus (Supplementary Fig. S2). LOC103949685 was annotated as Enhanced Disease Resistance 2-like in the public database because of its sequence homology with *Arabidopsis* EDR2 (At4g19040). Moreover, the sequences showed that the former protein is composed of 306 amino acids, while the latter has 720 amino acids, with significant differences in the homologous region. The 6-Asp-tandem repeat of *LOC103949685* allele A was conserved in the russet fruit skin samples, as revealed by DNA sequencing of 49 different pear varieties (Supplementary Table S5), suggesting that *LOC103949685* allele A may control the russet trait of fruit skin.

### Expression profile of *LOC103949685* in pear tissues

**Figure 3:**
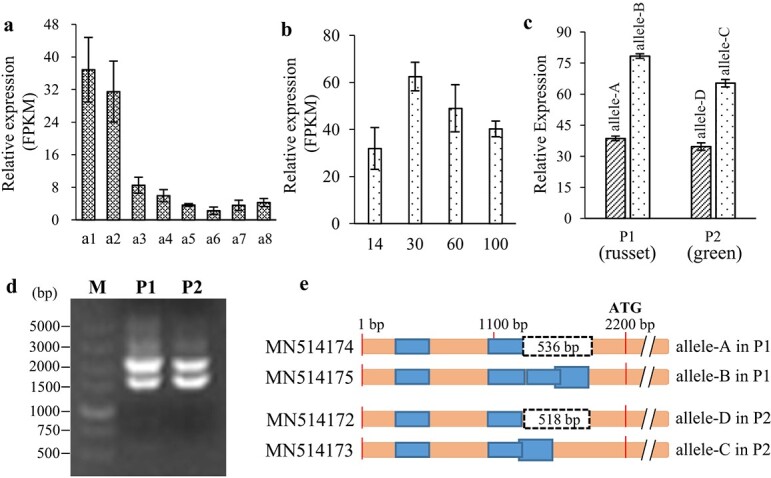
Promoter and expression characteristics of *LOC103949685*. **a** and **b** Relative expression levels of *LOC103949685* differed across tissues and developmental stages. In **a**, columns a1–a8 represent (a1) russet fruit skin; (a2) green fruit skin; (a3) flesh of russet skin fruit; (a4) flesh of green skin fruit; (a5) periderm of the biennial branch; (a6) periderm of the annual branch; (a7) root periderm; and (a8) leaf. **b** Numbers 14–100 below the columns represent russet fruit skin at 14, 30, 60, and 100 days after anthesis. **c** Expression differences between parental alleles in fruit skin samples. The mRNA-seq data of different sand pear tissues were analysed. The reads containing each typed SNP between pairs of alleles (allele A vs allele B; allele C vs allele D) were estimated as the relative expression of each allele. **d** Electrophoresis of PCR products obtained using universal promoter primers for *LOC103949685* alleles and the genomic DNA of each parent as the template. **e** Schematic of promoter sequence alignment. Sequence deletion is indicated by a dashed box. Promoters identified by the online program Transcription Start Site Prediction (TSSP, Prediction of Plant Promoters) [32] are shown as blue boxes. P1, parent cv. ‘Qingxiang’; P2, parent cv. ‘Cuiguan’. All promoter sequences are deposited in the NCBI database with accession numbers MN514172–MN514175.

RNA-seq revealed that the fruit skin showed higher expression of *LOC103949685* compared with other sand pear tissues (Fig. 3a), and the expression profile in fruit skin showed a peak at 30 days after anthesis (DAA) and then decreased with fruit development (Fig. 3b). Additionally, the expression of allele A in russet fruit skin was similar to that of allele D in green fruit skin, which was approximately half that of allele B in russet skin samples or allele C in green skin samples (Fig. 3c). Cloning and alignment of the gene sequences showed that a segment containing a predicted promoter was lost in the promoter regions of both allele A and allele D compared with that of allele B and allele C (Fig. 3d and e).

### Confirming the PCD function of allele A of *LOC103949685*

**Figure 4:**
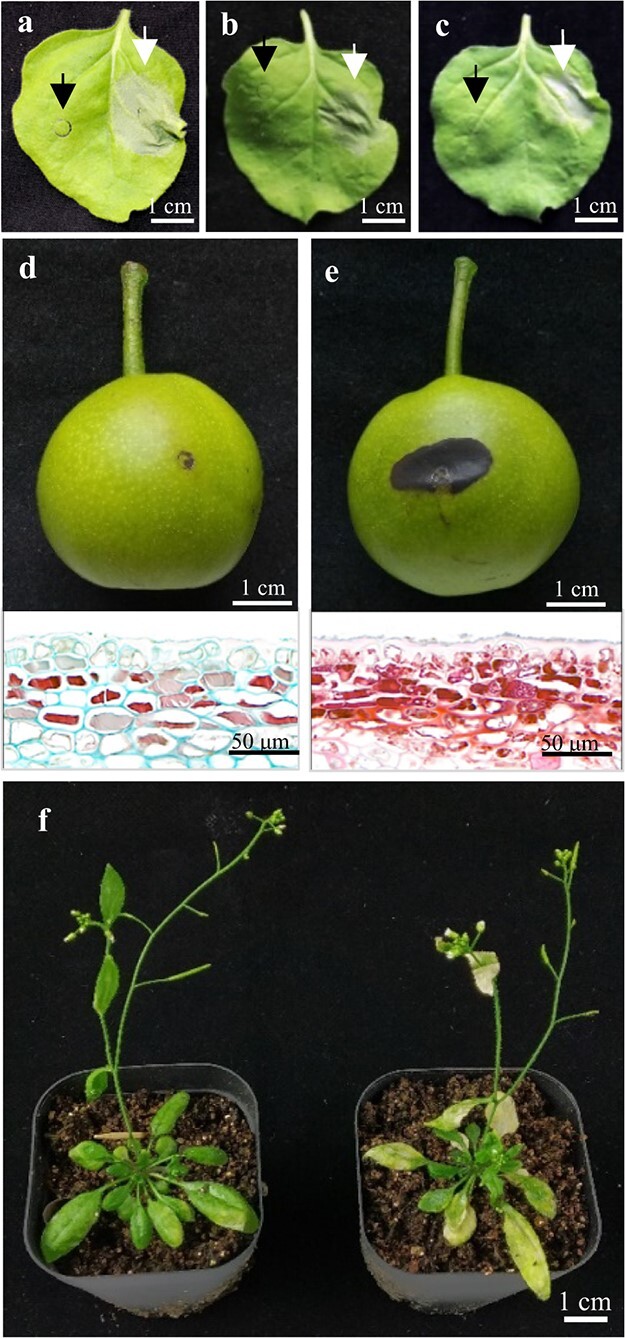
Lethal function of PyPPCD1.1 in plants. **a**–**c** Expression of *Pyppcd1.1* in *N. benthamiana*, with the control indicated by a black arrow. **a***PyPPCD1.1* is indicated by a white arrow. **b** The (Asp)6 mutation in the C-terminus of Pyppcd1.1 is indicated by a white arrow. **c** The Asp repeat deletion mutation of Pyppcd1.1 is indicated by a white arrow. **d** and **e** Expression of *Pyppcd1.1* (D) and *PyPPCD1.1* (E) and *Pyppcd1.1* (left) in the green skin fruit of *P. pyrifolia* with the corresponding microscopic view. **f** Expression of *PyPPCD1.1* (right) and *Pyppcd1.1* in *Arabidopsis* under the control of the oestradiol-induced promoter by spraying the 20-day-old whole plant with 20 μM oestradiol.

Since PCD is involved in russet formation of pear fruit skin, the potential function of allele A was first analysed using its transient expression in *Nicotiana benthamiana* leaves. As expected, this allele triggered severe injury and death around the injection site on the leaf (Fig. 4a) and produced necrotic spots at the infection site on pear green fruit skin (Fig. 4e). A similar result was observed in russet fruit skin and at the necrotic spot of green fruit skin by transient expression of allele A (Fig. 4e). The oestradiol-induced promoter was selected as the expression control in transgenic plants due to the lethal effect of allele A. The leaves of *T*_1_ transgenic *Arabidopsis* showed obvious loss of green colour and death after spraying with 20 μM oestradiol for 3 days (Fig. 4f). The effects of (Asp)6 mutations at the C terminus or the deletion of Asp repeats from allele B were also detected by transient expression in *N. benthamiana* leaves, both of which produced global necrosis, similar to that of allele A, around the injection site (Fig. 4b and c). The lethal function of allele A explained the PCD observed in russet fruit skin, as PCD could produce the initial signal for complex regulation and periderm suberization. Mutations that decreased the number of Asp repeats at the C terminus conferred the lethal function of the russet gene with up to six Asp repeats based on *LOC103949685* alleles. Considering other alleles with PCD function, we named allele A *PyPPCD1.1* (*Pyrus Periderm Programmed Cell Death 1.1*). As they are recessive to *PyPPCD1.1* in russet pear fruit skin, alleles B, C, and D of *LOC103949685* were named *Pyppcd1.1*, *Pyppcd1.2*, and *Pyppcd1.3*, respectively.

### Functional conservation analysis of PyPPCD1.1 homologues

**Figure 5:**
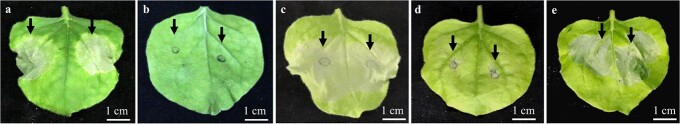
Phenotype of *PyPPCD1.1* homologue expression in *N. benthamiana* leaves (**a**–**c**) and *PyPPCD1.1* coexpression with *ARF1*-RNAi in *N. benthamiana* leaves (**d**, **e**). **a***N. benthamiana* leaf expressing *P. trichocarpa LOC7465446*. **b***N. benthamiana* leaf expressing *A. thaliana AT5G10410*. **c***N. benthamiana* expressing a mutant fragment where the 3′-end of *AT5G10410* was replaced with the coding sequence from a segment of *PyPPCD1.1* (marked brown in Supplementary Fig. S3). **d***N. benthamiana* expressing *PyPPCD1.1* and *ARF1-RNAi*. **e***N. benthamiana* expressing *PyPPCD1.1* and the empty plasmid. In **d** and **e**, both *ARF1*-RNAi and the empty vector were inoculated 48 hours prior to *PyPPCD1.1*. All images were captured at 72 hours after the last injection.

Querying PyPPCD1.1 against the NCBI reference protein database (ref_seq protein) yielded 61 unique PyPPCD1.1 homologues from 40 plant species: 34 eudicots, 3 gymnosperms, and 3 monocotyledons. Protein sequence alignment showed that PyPPCD1.1 homologues had two highly variable regions at the two ends of the protein (Supplementary Fig. S3). In the phylogenetic analysis, the differentiation of PyPPCD1.1 homologues was related not only to the relative distance of the species but also to differences between the woody/perennial and the herbaceous/annual, while the homologues in the monocotyledons and gymnosperms showed a higher divergence compared with those of the eudicots and were distributed as the outgroups of the tree (Supplementary Fig. S4). To analyse the effect of sequence differentiation among the PyPPCD1.1 homologues on protein function, we selected two genes from *Arabidopsis thaliana* and *Populus trichocarpa* for transient expression in *N. benthamiana* leaves. The results showed that the PyPPCD1.1 gene from *P. trichocarpa* has high variation in the N-terminus but is conserved in the C-terminus; this feature triggers PCD in *N. benthamiana* leaves. However, the PyPPCD1.1 homologue in *A. thaliana*, which had high variation at both ends of the protein, failed to trigger PCD in *N. benthamiana* leaves (Fig. 5a and b). When the mutant at the C terminus of the *Arabidopsis* homologue was replaced with a segment of PyPPCD1.1 (marked in Supplementary Fig. S3) and transferred to the *N. benthamiana* leaf, apoptosis occurred around the injection site (Supplementary Fig. 5C). These findings indicated that the segment of the PyPPCD1.1 homologue containing the C-terminal variation is directly related to the PCD protein.

### Subcellular localization variation between PyPPCD1.1 and Pyppcd1.1

**Figure 6:**
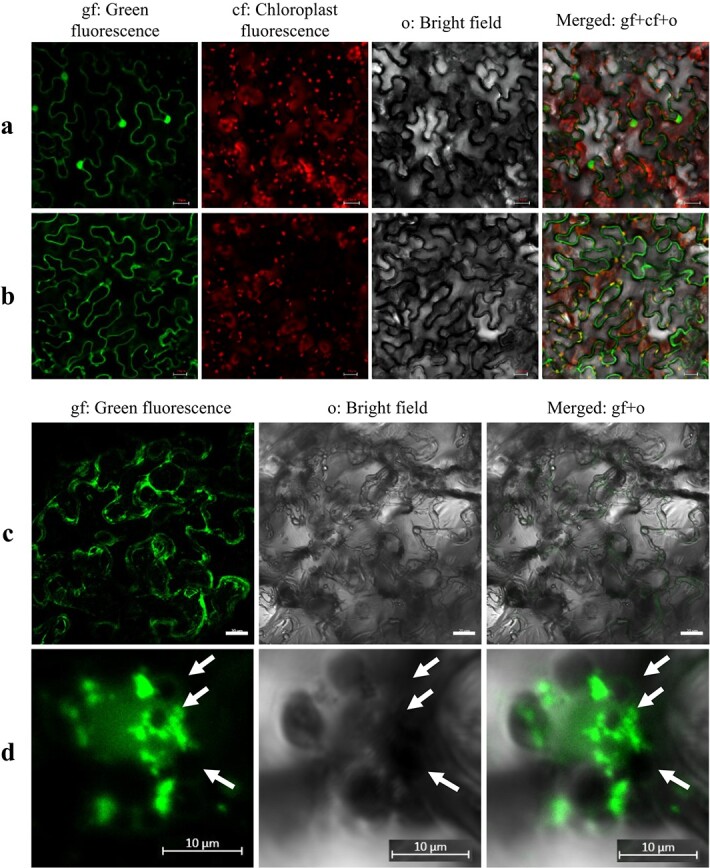
Subcellular localization of PyPPCD1.1 and Pyppcd1.1. **a** and **b** Pyppcd1.1-GFP and PyPPCD1.1-GFP 22 hours after injection in *N. benthamiana* leaves. **c** and **d** PyPPCD1.1-GFP 72 hours after injection in *N. benthamiana* leaves, at various magnifications. Arrows in **d** indicate putative holes or bubbles created during the collapse of the nuclear membrane (scale bars: **a** and **b**, 50 μm; **c**, 20 μm; **d**, 10 μm).

Subcellular localization analysis by Green Fluorescent Protein (GFP) fusion revealed that Pyppcd1.1 was distributed in the nucleus, plasma membrane, cytoskeleton, and cytoplasm (Fig. 6a). Conversely, the fluorescence signal of the PyPPCD1.1-GFP fusion protein was weak in the nucleus and cytoskeleton but was detected in the separated chloroplasts, and similar to the control (Fig. 6b); this expression became diffuse along the plasma membrane as the membrane system collapsed and nuclear fragmentation occurred during cell death (Fig. 6c and d). PyPPCD1.1 promoted increased production of vesicles at the plasma membrane at the late infection stage (Fig. 6c). All the homologues from *P. trichocarpa* and *A. thaliana* and the mutant showed subcellular localization similar to that of PyPPCD1.1 fused with GFP (Supplementary Fig.S5).

### Identifying the interaction between PyPPCD1.1 and ADP ribosylation factor 1

**Figure 7:**
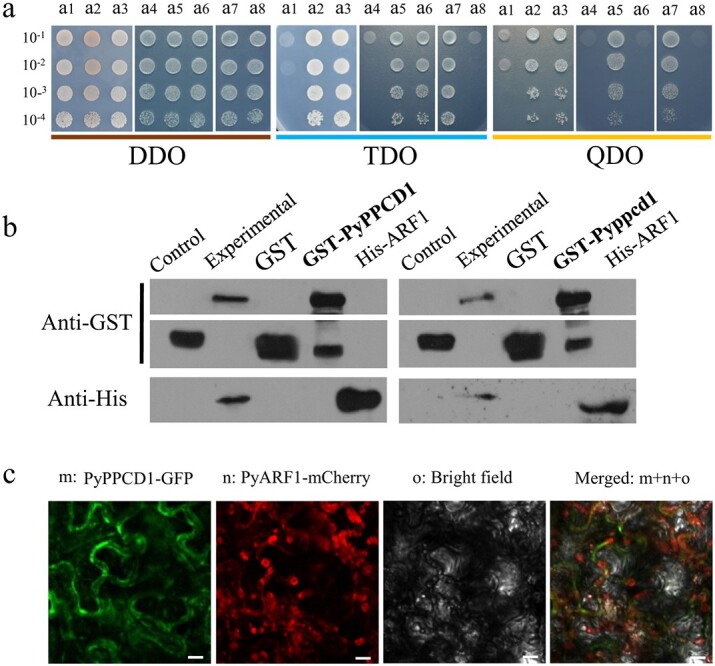
Interaction and subcellular colocalization analysis of PyPPCD1.1/Pyppcd1.1 and PyARF1. **a** Analysis of the interaction between PyPPCD1.1 and PyARF1 and the interaction between Pyppcd1.1 and PyARF1 by Y2H assays. Yeast strain NMY51 was cotransformed with the indicated plasmid pairs and spotted onto DDO, TDO, and QDO media in a 10-fold dilution series. All clones grew well on TDO and/or QDO media, indicating the interaction of both PyPPCD1.1 and Pyppcd1.1 with PyARF1. (a1) pBT3-N-Pyppcd1.1/pPR3-N; (a2) pBT3-N-Pyppcd1.1/pPR3-N-POST-Nubal; (a3) pBT3-N-Pyppcd1.1/pPR3-N-PyARF1; (a4) pBT3-N-PyPPCD1.1/pPR3-N; (a5) pBT3-N-PyPPCD1.1/pPR3-N-POST-Nubal; (a6) pBT3-N-PyPPCD1.1/pPR3-N-PyARF1; (a7) pTSU2-APP/pNubG-Fe65 (positive control); and (a8) pBT3-SUC/pPR3-N (negative control). The pBT3-N-Pyppcd1.1/pPR3-N-PyARF1 pair (a3) had good growth, but the pBT3-N-PyPPCD1.1/pPR3-N-PyARF1 pair (a5) was repressed on QDO medium, suggesting that the interaction between PyPPCD1.1 and PyARF1 was weaker than that between Pyppcd1.1 and PyARF1. **b** Interaction between PyPPCD1.1 and ARF1 and that between Pyppcd1.1 and PyARF1 by pull-down analysis. GST-tagged PyPPCD1.1, GST-tagged Pyppcd1.1, and His-tagged ARF1 were detected by GST and His antibodies. **c** Subcellular colocalization analysis of PyPPCD1.1 and PyARF1 72 hours after injection in *N. benthamiana* leaves. Scale bar, 20 μm.

To explore the potential mechanism underlying PyPPCD1.1 function and how the decrease in Asp repeats affects protein function, we analysed the protein structure by homology search using the I-TASSER online platform [33, 34]. The putative homologous protein comprises numerous regularly arranged subunits or capsomeres that contribute to the structural integrity of the complex or its assembly within or outside the cell. Notably, the top 10 identified structural analogues of PyPPCD1.1 belong to viral coat proteins (Supplementary Table S6). The pear fruit skin cDNA yeast two-hybrid (Y2H) library was screened using the Pyppcd1.1 open reading frame (ORF) as a bait for potential protein interactions. The results identified a strong interaction between Pyppcd1.1 and an ADP ribosylation factor 1-like protein (LOC103963967, termed PyARF1); conversely, the interaction was weak between PyPPCD1.1 and PyARF1 (Fig. 7a). The direct interactions between both PyPPCD1.1 vs ARF1 and Pyppcd1.1 vs ARF1 were confirmed by GST pull-down assays (Fig. 7b).

For the transient expression analysis in *N. benthamiana* leaves, PyPPCD1.1 showed subcellular colocalization with PyARF1 in the plasma membrane and cytoplasm (Fig. 7c; Supplementary Fig. S6C and D). When the sequence was compared against the pear reference genome, another 11 ARF1 homologues with 41.6–99.4% identity to PyARF1 were identified (Supplementary Fig. S7A); however, PyARF1 showed high expression levels for both transcripts and proteins compared with the other homologues in each type of fruit skin, and the ARF protein levels in russet fruit skin were significantly higher than those in green fruit skin (Supplementary Fig. S7B and C).

### PyPPCD1.1 triggers PCD in an ARF1-dependent manner

To investigate whether ARF1 is involved in triggering PCD by PyPPCD1.1, we cotransformed *PyPPCD1.1* with the *ARF1* RNAi vector. The results showed that *PyPPCD1.1* could not initiate PCD when injected into *N. benthamiana* leaves (Fig. 5d). This finding suggested that ARF1 is essential for PyPPCD1.1-guided cell death. PyARF1 belongs to the class-I ARF GTPases, which are conserved in eukaryotes as a critical part of the machinery responsible for vesicle scission, which induces vesicle fission through amphipathic helices upon dimerization [35–38]. The overexpression of *NbARF1* can directly kill *N. benthamiana* cells, and ARF1 guanosine triphosphatase (GTPase) can induce cell death [30]. Since the membrane scission can cause large amounts of damage, ARF function is tightly regulated by functional networks [39]. These results suggested that ARF1 is a binding partner of PyPPCD1.1 with a potential role in ARF1 functional regulation. The heterologous expression of *PyARF1* in tobacco leaves produced vesicle-like structures at the plasma membrane but did not lead to membrane collapse or cell death within 3 days after infiltration (Supplementary Fig. S6B). In *PyPPCD1.1* transient expression in *N. benthamiana* leaves, ruptured, small, dense vesicle-like structures were observed at the plasma membrane (Fig. 6c and d).

## Discussion

Periderm is ubiquitous, covering the root and stem of most woody eudicots and gymnosperms, as well as some reproductive organs, such as the fruit of pear and kiwi. The development of the periderm of various organs and plant species was linked to the expression of suberin-related genes and cell death. During the development of the periderm in the pear fruit skin, phellogen first arises from the subepidermal layer (Fig. 1), which is common in the stem of most trees [14, 40]. Notably, the cells were similarly enlarged at the corresponding position of both russet and green fruit skin (Fig. 1); these enlarged cells can increase the extensibility of the fruit skin to accommodate the effects of the biaxial tangential strain and stress produced by the increase in fruit volume.

The different development degrees of the periderm make pear fruit skin show either partial or full russeting, which could be analysed by gene mapping as a quantitative trait locus (QTL)-controlled trait. A conservative major QTL at the end of linkage group 8 has been revealed in previous studies [41–44]. According to their different genetic characteristics, we typed partial russet and full russet as two separate traits; the former is a quantitative trait susceptible to environmental stresses, while the latter is a qualitative trait controlled by a dominant gene [26]. Thus, the study of russet mapping was simplified, and the full russet cosegregation markers obtained in this study showed a corresponding position in the previous major QTL at the end of linkage group 8, which was conserved in the different pear genotypes or cultivars (Supplementary Table S5). The nature of motif repetition makes SSRs inherently unstable and prone to mutation by mechanisms of replication slippage or unequal crossing over [45]. The change in the repeats for the SSR (GAC)_n_GAT in the *LOC103949685* coding sequence leads to coding products with increased or decreased Asp repeats, which correspond to the different functional alleles. The expression of *PyPPCD1.1* efficiently triggered cell death in both pear fruit skin and tissues undergoing heterologous expression, suggesting that the mechanism responsive to *PyPPCD1.1*-guided cell death is common in these plant species. The fruit skin expression and protein turnover results suggested that *PyPPCD1.1* was under combined selection at both the protein structure and gene expression levels during evolution. The temporal and spatial expression patterns of *PyPPCD1.1* homologues could also be selected to control PCD and even periderm formation of specific tissues at their developmental stages. Although rare compared with that in woody plants, the periderm also exists in some organs of herbaceous plants, such as in *Arabidopsis* roots and hypocotyls [13]. DNA methylation and histone modifications play a role in cork differentiation and chromatin remodelling associated with cell death in the young and traumatic periderms of cork oak [46]. A vacuolar processing enzyme in potato was revealed to be translocated to the vacuole through the autophagy pathway to induce cell death [47], while the GRAS transcription factor PtSHR2B played a role in phellogen formation and could regulate phellem and periderm formation [48]. The functional loss of the *PyPPCD1.1* homologue in *Arabidopsis* suggested that plants could also employ other genes or different molecular regulatory networks with a similar role to that of *PyPPCD1.1*.

Suberization occurred earlier than cell death during periderm development in pear fruit skin (Fig. 1). However, the regulatory correlation between suberization and PCD is unclear. Notably, many PCD markers are expressed in the early stages of suberin deposition [13]; hence, PCD signals are crucial in the early regulation of suberin biosynthesis, and cell death is the final result of the PCD program. The current study showed that PyPPCD1.1 induces cell death in *N. benthamiana* leaves without suberization. Moreover, heterologous expression of a set of genes from the suberin pathway changed the accumulation of suberin in transgenic plants but did not redirect the original plant cell cycle to rapid cell death [22, 49–52]. Based on these results, we speculated that suberization is not required to trigger cell death during periderm development. Environmental stresses, including dehydration and mechanical damage, could induce periderm formation, replacing the primary fruit skin. Russet skin fruit has no cuticle covering (Fig. 1); its rate of water loss is significantly higher than that of green skin fruit, and the expression of dehydration response genes is induced [15, 17, 27]. Thus, we speculated that during the development of russet skin fruit, the water loss left developing periderm cells in a constant state of physiological dehydration, and the stress signal mediated the accumulation of suberin and other metabolites that contributed to later periderm formation [16, 53]. In addition, stress-related signals produced during the PCD process may also be involved in the regulation of suberin biosynthesis by secondary metabolism in adjacent cells.

Furthermore, Pyppcd1.1 has multiple roles in the structure and function of the cytoskeleton and membrane system. On the other hand, the decrease in Asp repeats hinders protein entry into the nucleus, alters the integrity of the cytoskeleton and membrane system, and leads to cell death. ARF1 is essential for PyPPCD1.1-guided cell death, during which PyPPCD1.1 exists in combination with ARF1 on the membrane, leading to membrane scission and vesicle-like structure formation (Fig. 6c). Previously, the plant ARF machinery was shown to affect recycling endosomes, endocytosis, and trans-Golgi networks and to be directly involved in cell death signalling [28, 54–56]. Plasma membrane-localized ARF6 influences protein sorting, endocytic pathways, and membrane structure [57, 58]. Moreover, ARF1 GTPase functions as a signal transducer by activating signalling molecules in the MAPK pathway [59–61]. Additionally, MAPK signalling is involved in plant PCD regulation [62–65]. Thus, MAPK cascades were affected downstream of PyPPCD1.1-guided cell death.

As a major component of biological membranes, lipids serve as substrates for generating numerous signalling molecules that mediate the regulation of multiple biological processes, including biotic and abiotic stress responses and cell death [66–71]. ARF1 activates phospholipase D [72, 73], interacts with phosphatidylinositol 4-phosphate 5-kinase [65, 66], and promotes the production of phosphatidic acid and phosphatidylinositol 4,5-biphosphate. The expression of *PyPPCD1.1* alters the structure, disrupts the plasma membrane (Fig. 6c; Supplementary Fig. S6D), and regulates lipid biosynthesis, metabolism, and oxidation–reduction [17, 27, 74].

Compared with slow senescence during the plant ageing process, PyPPCD1.1 causes rapid and intense cell death (Fig. 4), similar to necrosis in animal PCD [75] and PCD in the plant immune response [76]. In addition, our previous studies revealed that PyPPCD1.1 also triggered the responses of a large set of disease-related genes at both the mRNA and protein levels in russet fruit skin [17, 27, 74], including *GLP* genes (GI: 103933808, 108865350, 103941350, and 103963620), *RPM1*-like genes (GI: 103962513), and *RGA2*-like genes (GI: 108867148), which were significantly upregulated in russet fruit skin [74]. ARF1 is involved in infection by plant pathogens, and membrane modulation by ARF1 complex capture of host cells facilitates pathogenic invasion in both plants and humans [30, 31]. ARF1 has also evolved as a functional molecule in the host to initiate the response of non-host resistance and R-gene-mediated resistance [28, 29]. These findings suggested that the mechanism underlying the developmental PCD in periderm cork formation shares core effectors with the PCD induced by pathogenic invasion. PyPPCD1.1 shears a DUF1336 domain with edr2, which plays a fundamental role in the cell death for plant defensive response [77]. DUF1336 domain can take a conservative role in regulating different types of cell death in plant.

Since the periderm acts as the first line of defence for a plant and as a source of phellem, it is crucial in crop stress resistance and cork production [2–4, 11, 13]. Thus, as the periderm-formation-determining gene, *PyPPCD1* is crucial for biotic and abiotic stress resistance and cork production. In addition to its protective function, the periderm in fruit skin is also related to fruit appearance and quality. Russeting caused by partial periderm formation is a commercially important defect in the skin of pear and apple [15, 26]. This study identified the gene controlling the russet trait of fruit skin, which could be used in fruit breeding for high quality appearance. The detailed roles of *PyPPCD1* homologues in different tissues across different species need to be explored in the future.

## Materials and methods

### Plant materials

All pear materials were cultivated at the Yangdu orchard of the Zhejiang Academy of Agricultural Sciences (30°82$^{\prime} $90$^{\prime\prime} $ N, 120°81$^{\prime} $50$^{\prime\prime} $ E, Haining County) in Zhejiang Province, China. A total of 1129 *F*_1_ individuals from 12 populations segregating for russet fruit skin were used to analyse the genetic characteristics of the russet trait in pear fruit skin (Supplementary Table S1). A population containing 255 *F*_1_ progeny from a cross of cv. ‘Qingxiang’ (russet skin) and ‘Cuiguan’ (intermediate skin) were used for linkage analysis of the russet gene. A total of 49 pear varieties with known fruit skin colours were used in the association analysis of phenotypes with molecular polymorphisms at the russet gene locus. All gene expression analysis materials were prepared according to the protocols described previously [17, 27, 74].

### Embedding and histological staining of pear fruit skin

Fruit-surface samples were prepared with a size of 10 × 5 × 5 mm^3^ and fixed in Formaldehyde-acetic acid-ethanol Fixative (FAA) for 24 hours. Then, dehydration in an ethanol–xylene series and Paraplast embedding were carried out according to standard methods described by Ruzin [78]. Tissue sections (4 μm) were stained with Safranin O/Fast green (Servicebio, Wuhan, China) to examine the tissue morphology. The sections were observed under a Nikon Eclipse E100 light microscope (Nikon, Tokyo, Japan), and images were displayed on a Nikon DS-U3 imaging system (Nikon).

### Nucleic acid extraction

DNA was extracted from unexpanded young leaves using a plant DNA kit (Tiangen, Beijing, China) according to the manufacturer’s instructions. RNA was extracted using the plant TRIzol kit (Invitrogen, Carlsbad, CA, USA).

### Linkage mapping

To map the russet gene of pear fruit skin, genomic DNA from 30 russet fruit skin and 30 green fruit skin samples was pooled. After constructing DNA libraries, Illumina sequencing (Illumina Inc., San Diego, CA, USA) was performed and sequence data were analysed using the pipeline protocol according to a previously described method [79]. The genomic data of Chinese white pear (deposited in the NCBI database) served as a reference for the alignment of sequencing reads. Comparison of RAD-tags was carried out within each pool and between the two DNA pools with two mismatches allowed. Then, the comparison results were integrated with read depth and yielded highly reliable SNP classifications for both DNA pools. As the russet trait of pear fruit skin is controlled by a dominant gene (Supplementary Table S1), only SNPs with a sequencing depth ratio meeting the χ^2^ test 1:1 criterion based on the russet skin pool were selected. However, those SNPs sharing the same variation at the corresponding sites in the green skin pool were filtered out in the subsequent gene mapping analysis. Chromosome enrichment analysis was carried out for all qualified SNPs, and the russet gene was mapped under the hypothesis that the trait is dominantly inherited. Fine mapping of the russet gene was carried out in the *F*_1_ population segregating for russet pear fruit skin. SSRs and SNPs (shown in Supplementary Tables S2 and S3) were selected based on the differences between the two parental genome sequences in the identified region at the end of linkage group 8. SNPs were detected using the Sequenom MassARRAY^®^ platform (Sequenom, San Diego, CA, USA), and SSR polymorphisms were detected by PCR-based polyacrylamide gel electrophoresis. The fruit skin colour was scored as a marker and combined with the available SNP and SSR markers for linkage map construction with Joinmap 4.0 [80] using a logarithm of the odds (LOD) score threshold of 5.0. Map distances were obtained based on the recombination values with the Kosambi function [81].

### Genome sequencing and allelic variation analysis

Genome sequencing of both parents was carried out on the Illumina HiSeq 2500 platform using the PE125 sequencing method after DNA library construction. Low-quality read filtration was performed before sequence alignment to the reference genome. According to the alignment results, sequence differences, including SNPs, small indels, and structural variations, were detected between the sample and the reference genome. In addition, allelic variation between the two parents was analysed based on alignment with the reference genome as a bridge. Structural variation in the genic region was highlighted by functional gene annotation, discrimination between the coding region and regulatory region, and synonymous and non-synonymous mutations.

### Gene cloning

Pear is a self-incompatible species with two sets of heterozygous chromosomes. The alleles at LOC103949685 were cloned from both genomic DNA and cDNA templates. To prepare the cDNA template, 5 g of fruit skin tissue was collected from each of the parents at 80 DAA and used for RNA extraction, and reverse transcription was carried out using the PrimeScript™ RT–PCR Kit (TaKaRa Bio Inc., Otsu, Japan). Gene-specific primers (F1 5′-ACAGGAGAGAGTCATAGTGCG-3′, R1 5′-GCTACCGCCGGTTAAGATT-3′) were used for PCR under the following conditions: 94°C (3 minutes); 35 cycles of 94°C (30 seconds), 58°C (20 seconds), and 72°C (2 minutes); and 72°C (5 minutes). The 50-μl reaction mixture consisted of 20 ng of template, 5 pmol of each primer, 20 nmol dNTPs, 37.3 nmol MgCl_2_, 0.5 U Pyrobest^®^ DNA Polymerase (TaKaRa), and 1× PCR buffer. According to the promoter prediction based on the reference genome sequence, the specific primers 5′-TGCACTCGTGTACTTAGGCA-3′ and 5′-AGTGGTGCTTCTCTACTCGT-3′ were designed to clone the 2000-bp sequence preceding the start codon of *LOC103949685*. PCR was carried out using the genomic DNA from each parent as the template, with the same polymerase and conditions as those described above. The PCR product was detected by 1% (w/v) agarose gel electrophoresis; the amplicons were purified using the TIANquick Midi Purification Kit (Tiangen, Beijing, China). Subsequently, the products were cloned into the pEASY-T1 vector and transformed into *Escherichia coli* Trans1-T1 phage-resistant chemically competent cells (TransGen Biotech Inc., Beijing, China). Positive colonies were verified by PCR using specific primers and then sequenced on the ROCH-454 sequencing platform. Structural variation between the alleles of each parent was analysed by sequence alignment of the obtained positive colonies.

### RNA-seq analysis for gene expression

RNA-seq analysis was carried out according to previously described protocols [17, 27, 74, 82]*.* Briefly, RNA-seq library construction was performed using the Illumina TruSeq™ RNA Sample Prep Kit. Each library underwent sequencing on an Illumina HiSeq platform and produced >6 Gbp of 150-bp paired-end original reads. After removing adapters and low-quality sequences, reads that met the quality requirements were mapped to the reference genome by Burrows–Wheeler alignment with Bowtie [83]. Only reads showing unique loci in the genomic sequence were applied for transcript abundance estimation to differentiate the expression among the alleles. Three biological replicates were assessed for each type of tissue/organ.

### Transient transformation in tobacco leaves and pear fruit

For transient transformation, *LOC103949685* alleles A and B were amplified using primers containing ASC1 or Pac1 restriction sites, respectively, and all the other genes and mutant alleles with these restriction sites were directly synthesized on a Dr. Oligo 48 Oligo Synthesizer (Biolytic^®^ Lab Performance, Inc., Fremont, CA, USA). Each of the PCR products and synthesized sequences was digested and inserted preceding the *GFP* or *mCherry* gene sequence in the expression cassette of the PTA7002 vector (constructed on the pCAMBIA0380 backbone) driven by the 2 × 35S promoter. For RNAi-induced silencing of gene expression, two 200-bp fragments from the tobacco *ARF1* (*LOC107770727*) coding sequence (bases 9–208) were amplified using primers. The amplicon was cloned between BsaI sites (*tgtF*-F 5′-cgatGGTCTCacaacgtctttcggcaaac-3′ and *tgtF*-R 5′-cgatGGTCTCactccaacatcccacacg-3′ for fragment 1, *tgtR*-F 5′-cgatGGTCTCagggcctccaacatcccacac-3′ and *tgtR*-R 5′-cagtGGTCTCatacagtctttcggcaaac-3′ for fragment 2). The 200-bp fragment for the loop structure was amplified from the intermediary vector using the primers loop-F 5′-cgatGGTCTCaggagcctgcaggtctagt-3′ and loop-R 5′-cgatGGTCTCagcccgggctctgtaactatc-3′. The amplification conditions for the PCR are mentioned above. The amplified fragment was digested with BsaI and cloned in sense orientation into pBWA(V)HS. After ligation to the vector and transformation into *E. coli* cells, the constructs were confirmed by sequencing. Subsequently, the constructs were transformed into *Agrobacterium tumefaciens* strain EH105 by the freeze-and-thaw technique, followed by growth in 0.5 ml LB broth at 28°C and 220 rpm for 4 hours. Then, the cells were cultured on LB agar containing 100 μg/mL Rifampicin (RFP) and 50 μg/mL kanamycin (BBI Co., Ltd, Shanghai, China) at 28°C for 2 days. Single colonies were cultured in 0.5 mL LB broth with the same antibiotic selection at 28°C and 220 rpm overnight and assessed by plasmid DNA extraction and PCR amplification of the target fragment. Positively transformed colonies were inoculated in LB broth medium containing 50 μg/mL kanamycin and 100 μg/mL RFP at 28°C to an OD600 of 0.6–0.8. Then, the bacteria were collected by centrifugation (3000 × *g*, 3 minutes) at ambient temperature, resuspended in MES buffer (10 mM MES, 10 mM MgCl_2_, 0.1 mM acetosyringone, pH 5.8) to an OD600 of 1.0, and incubated at 25°C for 4 hours without shaking before infiltration. The MS salt solution was used to dilute the bacteria to 30 times the original volume when applicable. Approximately 300 μl of the *Agrobacterium* suspension was inoculated into young *N. benthamiana* leaves or the flesh of young green skin fruit of sand pear (*Pyrus pyrifolia*). For subcellular colocalization analysis, the *Agrobacterium* mixture of PyPPCD1.1-GFP and PyARF1-mCherry was used to infect the tobacco leaves. Images of the infected leaves were acquired with a digital camera and a laser scanning confocal microscope at 24, 48, and 72 hours post-injection. Images of the infected fruits were obtained using a digital camera 1 week after the injection.

### Heterologous expression of the russet gene in *Arabidopsis*

To assess the lethal function of *PyPPCD1.1*, an oestradiol-induced promoter was selected for heterologous gene expression analysis in *Arabidopsis*. The ORF of *PyPPCD1.1* was amplified using the primers *pw2-10*-F 5′-cagtGGTCTCatttgatgtgtccaacaaagcaaaa-3′ and *pw2-10*-R 5′-cagtGGTCTCaagagtcaatcgtcgtcgtcgtcgt-3′, and the ORF of *Pyppcd1.1* was amplified with the primers *pw1-2*-F 5′-cagtCGTCTCatttgatgtgtccaacaaagcaaaa-3′ and *pw1-2*-R 5′-cagtCGTCTCaagagtcaatcgtcgtcgtcgtcgt-3′. The amplification conditions were as described above. The amplified fragment was digested with BsaI*,* cloned into the binary vector pBWA(V)HVE, and introduced into *Arabidopsis* (Ecotype Columbia-0), as described previously [84]. Then, *Arabidopsis* plants were grown as described by Cominelli *et al*. [52]. The effect of overexpression of these genes on the plant was assessed by spraying 25-day-old whole plants with 20 μM dexamethasone (Macklin, Shanghai, China).

### Protein model prediction

Protein structure and function were predicted by integrating comprehensive information on the structure, sequence, and protein–protein interactions in the I-TASSER server (https://zhanglab.ccmb.med.umich.edu/I-TASSER/) based on the homology modelling method [33, 34].

### Yeast-two-hybrid assays

A yeast two-hybrid (Y2H) library was constructed from pear fruit skin at the russet forming stage, according to the method described by Cao and Yan [85]. Briefly, cDNA was prepared by the switching mechanism at the 5′ end of the RNA transcript (SMART) method, followed by double-stranded cDNA ligation into the linearized pGADT7 cloning vector. This plasmid was introduced into yeast Y187 cells to construct the primary cDNA library. The bait recombinant plasmid pGBKT7-Pyppcd1.1 was cotransformed with the prey empty PGADT7 into the Y2H Gold yeast strain for toxicity and autoactivation analyses. The resulting cotransformants were first screened on SD/−Trp plates and further assessed for reporter gene expression on triple-dropout (TDO)/X-α-Gal plates. Positive yeast colonies with different interaction intensities were incubated in quadruple dropout (QDO)/X-α-Gal liquid medium. Then, the recombinant pGADT7 plasmids comprising the selected colonies were extracted and identified by sequencing. To test protein–protein interactions with PyPPCD1.1, toxicity and autoactivation assays of PyPPCD1.1 were carried out as described for Pyppcd1.1. Subsequently, the target plasmid combination was cotransformed with pGBKT7-PyPPCD1.1 into the Y2H Gold yeast strain and cultivated on synthetic double-dropout (DDO), TDO, and QDO plates. Cotransformation of the target plasmid and pGBKT7-PyPPCD1.1 was used as the control.

### GST pull-down assay

The coding sequence of *PyPPCD1.1*/*Pyppcd1.1* was linked to the GST tag in pGEX-4 T-3, and *PyARF1* was linked to the His tag in pBAD202 with gene-specific primers. Both recombinant vectors were transformed into BL21 (DE3) *E. coli* cells under ampicillin selection. Each colony was first inoculated in 5 ml of ampicillin-containing (AMP^+^) LB medium at 150 rpm and 37°C for 6 hours, followed by the addition of 200 ml AMP^+^ LB and 220 rpm shaking at 37°C until the OD600 reached 0.6–0.8. Protein overexpression was induced by 0.5 mM IPTG (pGEX-6p-1) or 0.02% arabinose (pBAD202) at 150 rpm and 18°C for 16 hours. The cells were harvested by centrifugation at 5000 × *g* and 4°C for 10 minutes. The cell pellet was resuspended in 1 ml of phosphate-buffered saline (PBS; KH_2_PO_4_ 2 mM, Na_2_HPO_4_ 10 mM, NaCl 137 mM, KCl 2.7 mM, pH 7.4) on ice with an ultrasonication processor (JY92-IIN) for 2 seconds with rest for 1 seconds until the suspension was transparent. After centrifugation of each cell suspension at 12 000 × *g* and 4°C for 50 minutes, the supernatant was mixed with dithiothreitol (DTT) at 1 mM. Then, an equivalent amount (0.5 mg) of GST-tagged fusion protein and His-tagged fusion protein were mixed and incubated on ice for 3 hours. Subsequently, the mixture was loaded onto glutathione Sepharose 4B resin columns. After washing five times with PBST (PBS with 0.3% Tween-20, v/v), proteins were eluted with wash buffer supplemented with 15 mM reduced glutathione. The eluates were separated using 12% sodium dodecyl sulphate–polyacrylamide gel electrophoresis (SDS–PAGE), transferred to polyvinyl difluoride (PVDF) membranes (Millipore, Billerica, MA, USA), and probed with anti-His and anti-GST antibodies (Sigma–Aldrich, Merck KGaA, Darmstadt, Germany). GST, GST-BrCRF6, His, and His-BrWUS1 from Wuhan Genecreate (Wuhan, China) were used as experimental controls. Three replicates were carried out for each pull-down assay.

### Collection and phylogenetic analyses of homologues

Homologues of PyPPCD1.1 were queried against the NCBI reference protein database using BLASTP [86]. Proteins were aligned using the CLUSTAL W algorithm implemented in the MegAlign package of the DNASTAR program (MegAlign 7.10, DNASTAR Inc., Madison, WI, USA). Phylogenetic analyses were carried out using MEGA software (version 4.0, http://megasoftware.net) using the neighbour-joining method with 1000 bootstrap replicates [87]*.*

## Supplementary Material

Web_Material_uhab061

## Data Availability

The following sequences can be found at GenBank database LOC103949685, LOC103963967, MN514172, MN514173, MN514174, MN514175, NC_003076 and LOC7465446. All data are available in the main text or the supplementary materials or in the databases previously named.
