# Supplementary material for: An ARF1-binding factor triggering programmed cell death and periderm development in pear russet fruit skin
Source: Hortic Res. 2022 Jan 19;9:uhab061. doi: 10.1093/hr/uhab061 (PMC8947239; doi:10.1093/hr/uhab061)
Supplement: Web_Material_uhab061 [file web_material_uhab061.zip › Fig. S1.pdf]

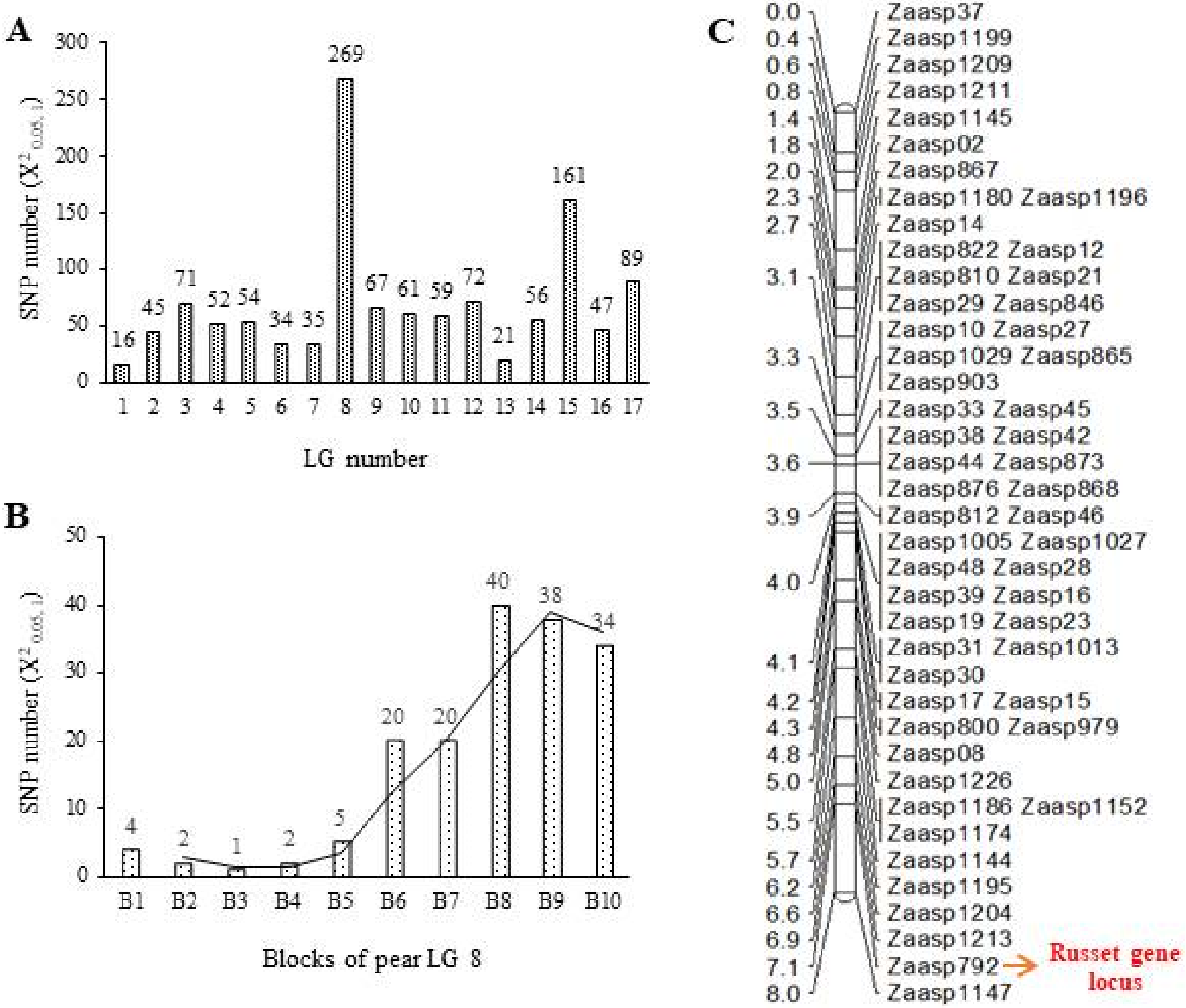


**Fig. S1.** Construction of a linkage map for the russet trait of sand pear fruit skin. A and B. chromosome distribution of the SNPs meeting the requirement of chi-square test. C. the linkage map for the russet trait of sand pear fruit skin. For chromosome mapping, SNPs were firstly localized to the scaffolds by sequence alignment, and then placed on the chromosome according to the scaffold mapping information by Wu et al. *(88)*. The map was constructed using the F1 progenies from reciprocal crosses of sand pear cv. Qingxiang and Cuiguan *(Table S1)* with 25 SNP markers *(Table S2)* and 32 SSR markers *(Table S3)*. The SSR marker *Zaasp792* showed co-segregation with the trait of russet in the assessed population.
