# Supplementary material for: An ARF1-binding factor triggering programmed cell death and periderm development in pear russet fruit skin
Source: Hortic Res. 2022 Jan 19;9:uhab061. doi: 10.1093/hr/uhab061 (PMC8947239; doi:10.1093/hr/uhab061)
Supplement: Web_Material_uhab061 [file web_material_uhab061.zip › Fig. S3.pdf]

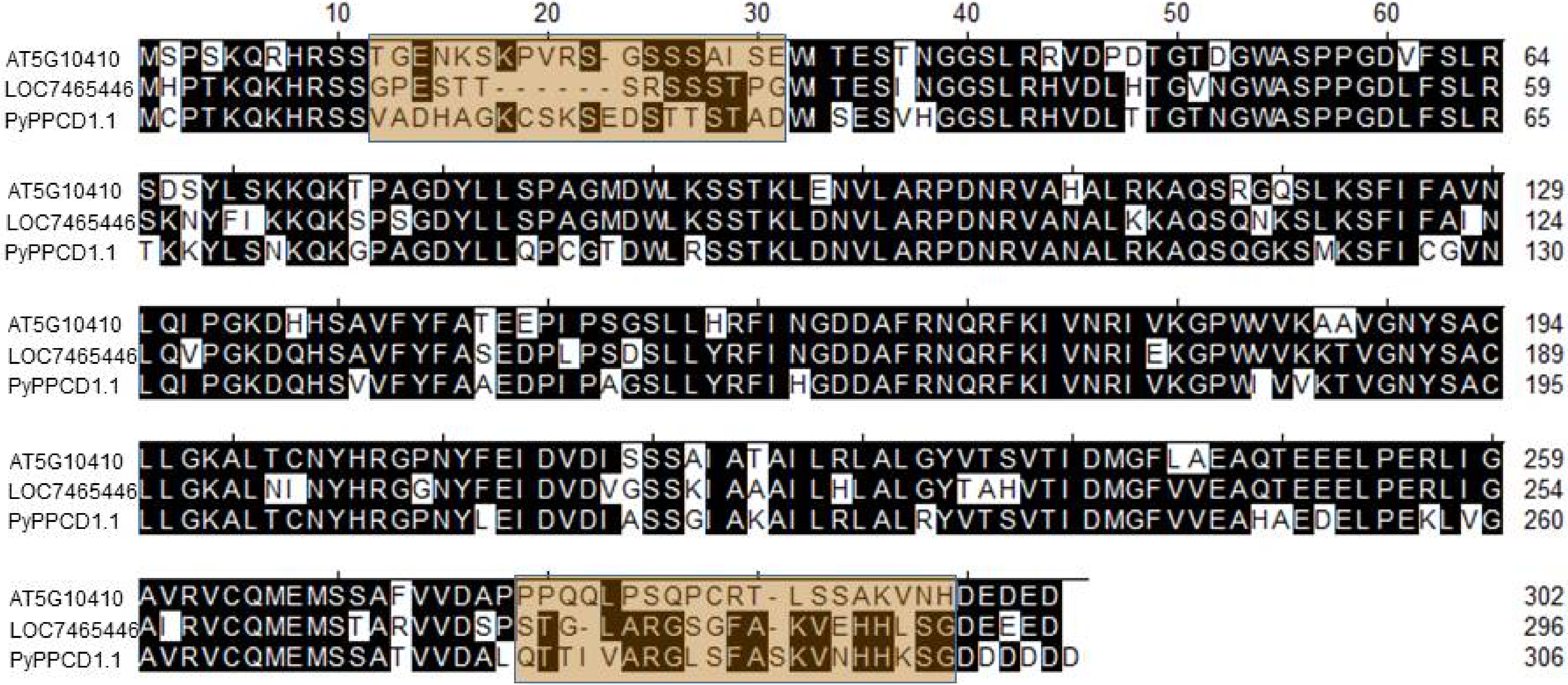

Fig. S3. Protein sequences alignment of PyPPCD1.1 and its homologues in *Arabidopsis thaliana* (AT5G10410) and *Populus trichocarpa* (LOC7465446). Shade (with solid black) residues that match the consensus exactly. High variation regions among the proteins were marked in brown.
