# Supplementary material for: An ARF1-binding factor triggering programmed cell death and periderm development in pear russet fruit skin
Source: Hortic Res. 2022 Jan 19;9:uhab061. doi: 10.1093/hr/uhab061 (PMC8947239; doi:10.1093/hr/uhab061)
Supplement: Web_Material_uhab061 [file web_material_uhab061.zip › Fig. S4.pdf]

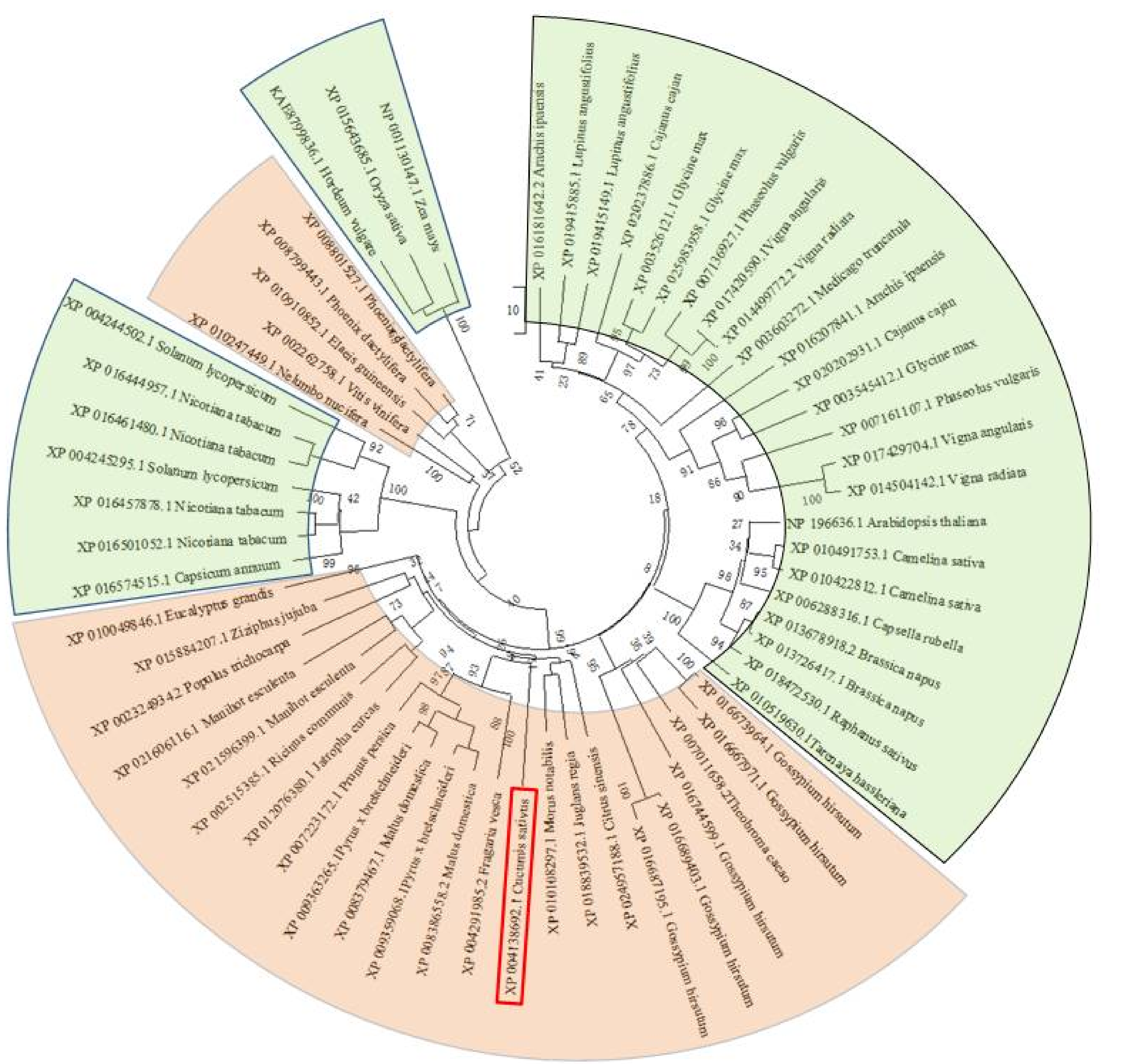

Fig. S4. The phylogenetic relationship of PyPPCD1.1 homologues. The tree was constructed based on multiple sequences alignment of LOC103949685 and its homologues from NCBI reference protein database using ClustalW program by NJ method with 1,000 bootstrap replicates. The homologues showed obvious differentiation between the perennial plants and annual plants, which were marked in brown and green, respectively. The exception from *C. sativus* was highlighted in red box.
