# Supplementary material for: An ARF1-binding factor triggering programmed cell death and periderm development in pear russet fruit skin
Source: Hortic Res. 2022 Jan 19;9:uhab061. doi: 10.1093/hr/uhab061 (PMC8947239; doi:10.1093/hr/uhab061)
Supplement: Web_Material_uhab061 [file web_material_uhab061.zip › Fig. S5.pdf]

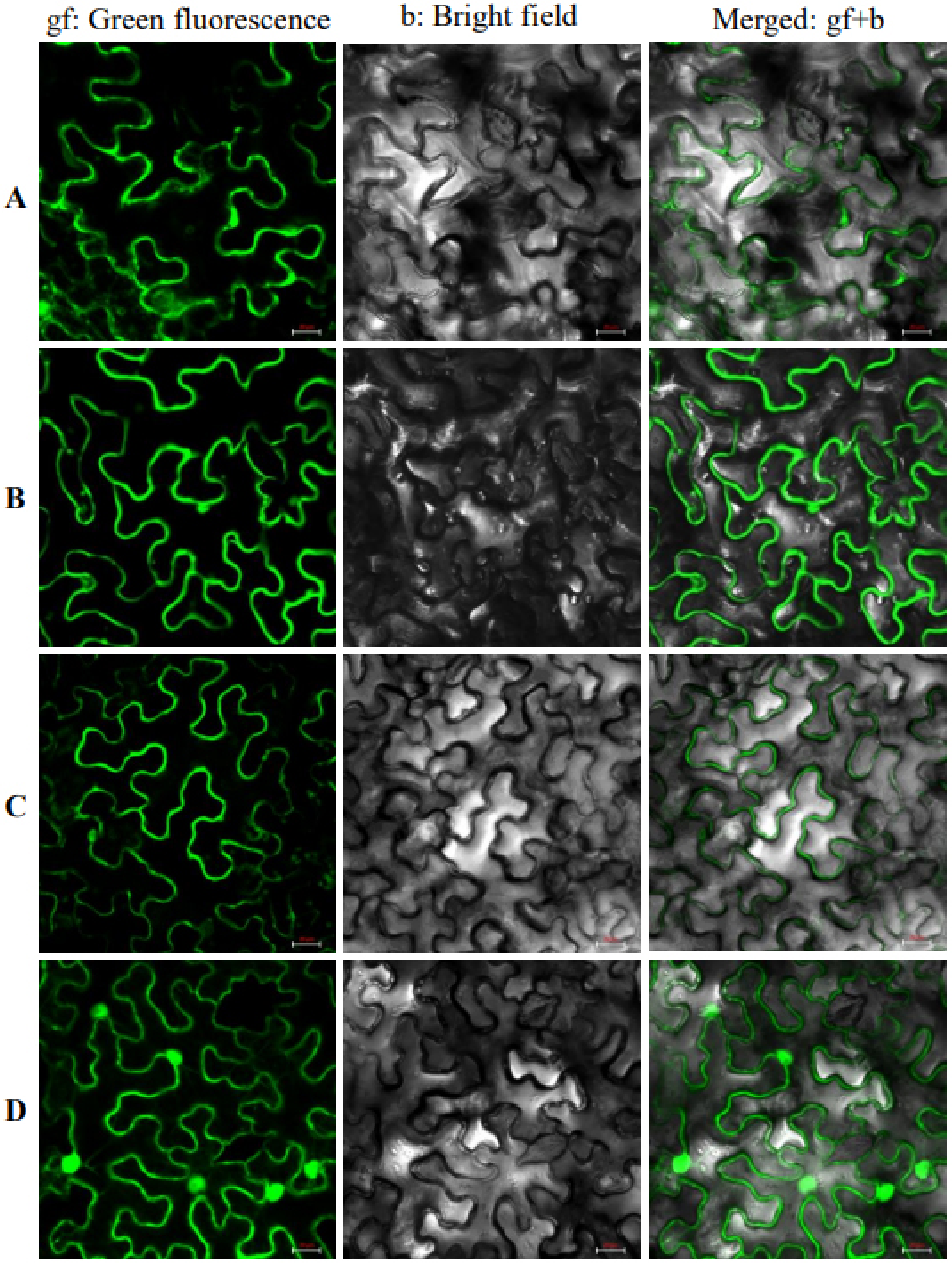

Fig. S5. Subcellular localization of PyPPCD1.1 homologs in *Populus trichocarpa*, *Arabidopsis thaliana* and the mutant of the *Arabidopsis* homolog. The mutant was derived from *AT5G10410* by replace its sequence at the CDS 3' end with the sequence of *the allele-A*, their corresponding amino acids marked in *Fig. S2*. A: LOC7465446, *Populus trichocarpa*. B: NC_003076, *Arabidopsis thaliana*. C: NC_003076 mutant. D: GFP Control.
