# Supplementary material for: An ARF1-binding factor triggering programmed cell death and periderm development in pear russet fruit skin
Source: Hortic Res. 2022 Jan 19;9:uhab061. doi: 10.1093/hr/uhab061 (PMC8947239; doi:10.1093/hr/uhab061)
Supplement: Web_Material_uhab061 [file web_material_uhab061.zip › Fig. S6.pdf]

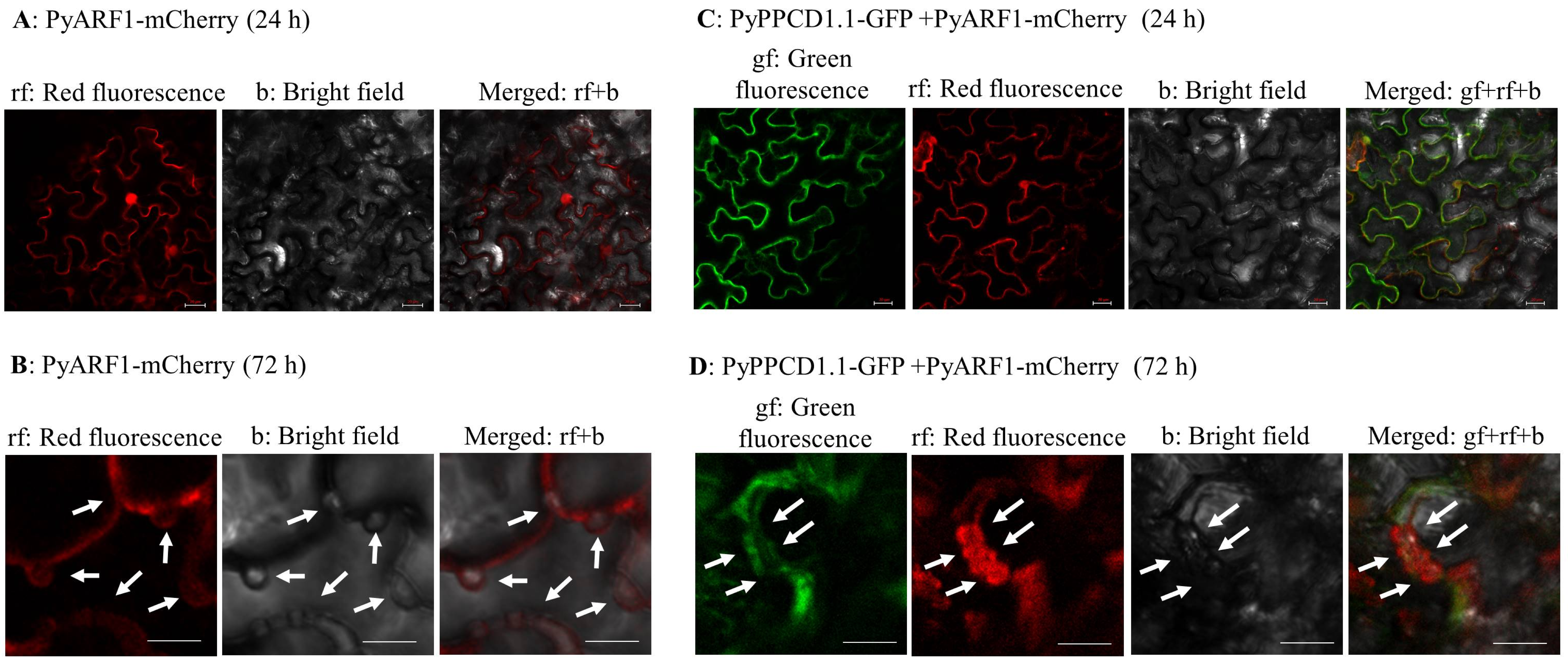

Fig. S6. Transient expression of *allele A* and *PyARF1* in *N. benthamiana* leaves. A and B. PyARF1 24 h and 72 h, respectively, after injection in *N. benthamiana* leaves. C and D. allele A colocalized with PyARF1 at 24 h and 72 h, respectively, after injection in *N. benthamiana* leaves. In B and D, the arrows indicate the putative circular or vesicle-like structure formed with plasma membrane scission mediated by *PyARF1* (red) individually or co-expressed with *allele A* (green) at 72 h after injection in *N. benthamiana* leaves (bars: 20 µm).
