# Supplementary material for: An ARF1-binding factor triggering programmed cell death and periderm development in pear russet fruit skin
Source: Hortic Res. 2022 Jan 19;9:uhab061. doi: 10.1093/hr/uhab061 (PMC8947239; doi:10.1093/hr/uhab061)
Supplement: Web_Material_uhab061 [file web_material_uhab061.zip › Fig. S7.pdf]

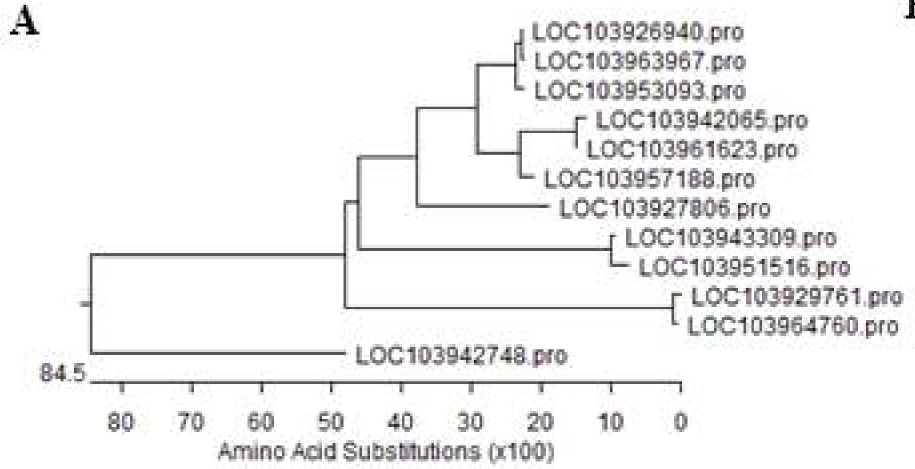

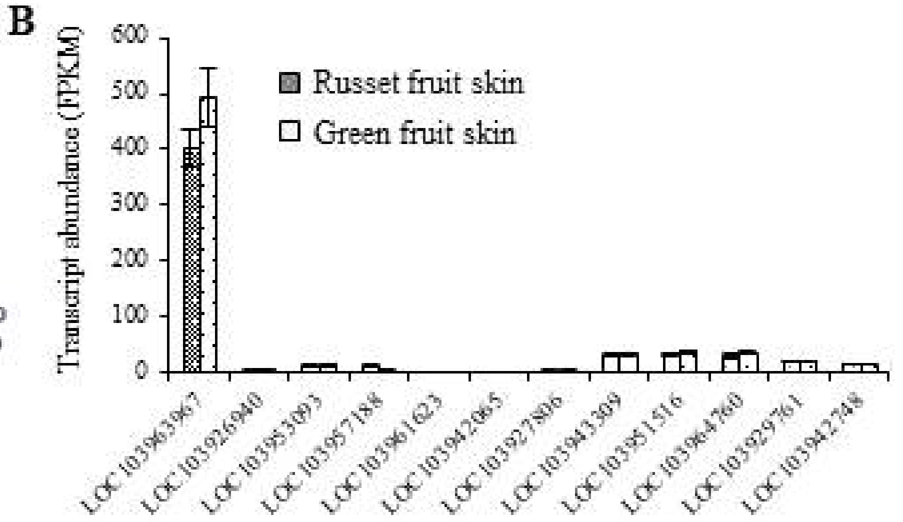

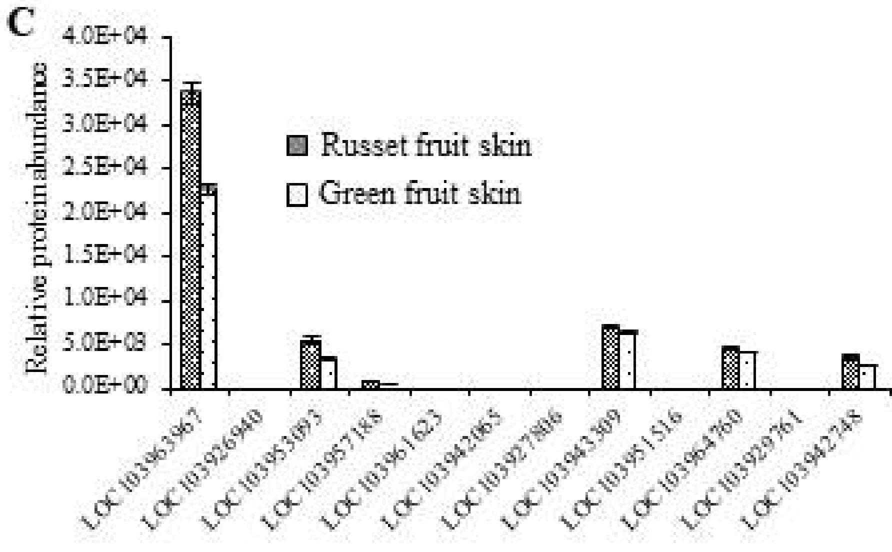

Fig. S7. PyARF1 homologues in pear genome and their expression in the russet and green fruit skin of sand pear. A. the phylogenetic tree of the pear ARF1 homologues constructed by MegAlign with Clustal W alignment method. B and C. the relative transcript and protein expression levels of the pear ARF1 homologs in the russet and green fruit skin of sand pear, derived from Wang et al. *(17)*.
