# Supplementary material for: An ARF1-binding factor triggering programmed cell death and periderm development in pear russet fruit skin
Source: Hortic Res. 2022 Jan 19;9:uhab061. doi: 10.1093/hr/uhab061 (PMC8947239; doi:10.1093/hr/uhab061)
Supplement: Web_Material_uhab061 [file web_material_uhab061.zip › Table S1.docx]

| **Cross:*♀×♂*(*****)** | **Fruit skin color** | | | ***X^2^*** （*X^2^*_0.05,1_=3.84） |
| --- | --- | --- | --- | --- |
|  | **Russet** | **Intermediate** | **Green** |  |
| ‘Akibae’×‘Cuiyu’(R, G) | 35 | 9 | 24 | 0.01 |
| ‘Yuguan’×‘Cuiguan’(R, I) | 32 | 0 | 19 | 2.82 |
| ‘Yuguan’×‘Cuiyu’(R, G) | 23 | 19 | 19 | 3.21 |
| ‘Yuguan’×‘Chuxialv’(R, G) | 41 | 22 | 14 | 0.21 |
| ‘Zaoshuhuangpi’×‘Cuiyu’(R, G) | 40 | 15 | 18 | 0.49 |
| ‘Akibae’×‘Chuxialv’(R, G) | 37 | 35 | 5 | 0.12 |
| ‘Qingxiang’×‘Cuiguan’(R, I) | 52 | 27 | 37 | 1.04 |
| ‘Cuiguan’×‘Qingxiang’(I, R) | 67 | 30 | 42 | 0.12 |
| ‘Cuiguan’×‘Za25’(I, R) | 37 | 18 | 20 | 0.00 |
| ‘Cuiguan’×‘Nikkori’(I, R) | 48 | 41 | 19 | 1.12 |
| ‘Cuiguan’×‘Akibae’(I, R) | 62 | 36 | 12 | 1.54 |
| ‘Cuiguan’×‘Zaoshuhuangpi’(I, R) | 92 | 61 | 21 | 0.47 |
| *Capital letters in the bracket denote the color abbreviation for the parent fruit skin. R=Russet, I=Intermediate or semi-russet, G=Green. | | | | |

**Table S1** Fruit skin russet segregation in the progenies of the twelve F1 populations of sand pear.
