# Supplementary material for: An ARF1-binding factor triggering programmed cell death and periderm development in pear russet fruit skin
Source: Hortic Res. 2022 Jan 19;9:uhab061. doi: 10.1093/hr/uhab061 (PMC8947239; doi:10.1093/hr/uhab061)
Supplement: Web_Material_uhab061 [file web_material_uhab061.zip › Table S2.docx]

| **Name** | **SNP type** | Sequence（85 bp） |
| --- | --- | --- |
| Zaasp02 | R(A,G) | AATTCTCAAATATTTCAATGTAGTTAACATTTGTGACTTTCCRTACAAGGCATGGTGGGACTATTAATATGTTTTGTAGCTTAAT |
| Zaasp08 | S(G,C) | AGTACTAACAGTTGATTAGTTATCTGTGGAGAATTCTGGTTTSTTGTCAATTTAGCTGGATATGAGATGTAATTGTTTTTTAGGG |
| Zaasp10 | W(T,A) | TTCGTATTTTGACAGAGATGTATGAATTCATATTGTAACTATWGCTTGGGAAAGGGCCAGCAGGAGAAACACAAACGAAAGGAGC |
| Zaasp12 | R(G,A) | AGATAATAATATTAAATTTTAAGTGAATTCATAACTTTACTCRGTGGTGTAATTCGTTGTCGCATTTTATTTTCATGAGTACCAT |
| Zaasp14 | S(C,G) | TATCCAATACTCTTCGATAGCTAGGAAACCGTTATTATATAAASGGAATATCAACATATATGTCAAGCAAAAAAGGCTTTCTTAC |
| Zaasp15 | Y(T,C) | CAATATACGACGAGGAGTTGAATTCTCGTATGCAAGGATGAAYGTCTTTGACCAATTGAAGTATATGTCATCTGTTATCACAACA |
| Zaasp16 | R(G,A) | TTGTGAATTCAAAATCTATACGCCGGCATGAATATAAAACCGRAAAGGTTTCATGACGGAAGAAATCTGCCGGCAGAGAAGGCAT |
| Zaasp17 | Y(C,T) | TACTGCGTACCCAAATTCCAGACGCCGTCTTCTGCCTCCCCTYTCGCTCTTCGCGGCGCTGCTACGAACTCCACTCTTCTTCGCG |
| Zaasp19 | S(G,C) | AGTGATTCACTGGGCCGCTCTCGGATTCGTGGACGGGAGGCTSTTCGGAAACCCTAAGTGCTTTCTTTAAGCGCTTCAGCTTCGT |
| Zaasp21 | Y(T,C) | ACGCGCTCACTTAGTAAAGTTCATGAACCATTCTCAAGCAAAYCAAAGATGTGTATTTTCAGTATAAATTAGCCGTAAACAGGCC |
| Zaasp23 | R(G,A) | TCCTCATTGAATTCATAAAATTATTTGTTCCGACCAATAAACRGGAAAATGTAAAATCGAATTTAAAAGCCCTAAGAAAATTAAT |
| Zaasp27 | R(A,G) | CAAATCAGGGAGAACCCTTTTGATATGCTGCTCCAGAATCTARCACAGAAAATCAACCATGACAACAGTGCAAAAACAACGAGAG |
| Zaasp28 | S(C,G) | AGGAATTCGTGCAAACATCGCACGAAAAAAAGTATATGTTCASAAGAAAATACTTGAAAACCTCGAAAAACACTAGACACTTACA |
| Zaasp29 | R(G,A) | GAAACATCACAGTTCTTCAAAATATGAAGAATTCTAACTTTCTRAGCAAATTCAGCTGACCTTGTGAATCGATCCGTCGTCGATA |
| Zaasp30 | M(A,C) | AATGTCAACCCTGAATTCAACGGCAATCGGCCATGAAAAAGGMAGAAGAAAAAAAATTAGGGTGAGACTTGTGAGCAAACCTTGA |
| Zaasp31 | Y(T,C) | AATATCTTTAACAAAGCATCAATTTTTGAGAATTCGACAACTYGTTCAAAGCAATCGAGGATTTTGGGAACGAAAGCAATCGAAG |
| Zaasp33 | R(A,G) | CTTTAGTGCCTCATAAGTGAATTCGTTACATTTGTTGTGAAARAATTTCATGGTGAACCCTCTTATTTATGTGCCTTTTTCTCGC |
| Zaasp37 | R(G,A) | ATTTACTAGACGATATTGTCATAAAAGGTATTGAATTCAGACRCATTGAAGAGCACAAACTATCCTGGCTAATTGACCTAACCCA |
| Zaasp38 | M(C,A) | ATTTTCTCCAACCAATAATGGAATTCAAAAACAAAGCGCATAMAATTCAAATCTCGACATCGGAAATCCACCACAAACAAGAGAA |
| Zaasp39 | Y(T,C) | TATGCAATAAAGCAGATCAGAATCAGCTCCAAAAAGAGACAAYAAAGTCAATGAACTATATGGTTCTAGCACCTACCTCCTCAAA |
| Zaasp42 | Y(C,T) | GAAGTACTGAAGTCCACATATTAGTATTAGGGTTACCAACTCYTTGCTAATAATAATATACTCACCCTTAGGAACAAAGAAGAGA |
| Zaasp44 | R(G,A) | AACTATAATGATATTGAATTCATCCATGGAAAACACCAAACTRTGAGTGGTGAAAACACTAACAAAGGAGTGGATAATTCGCTTA |
| Zaasp45 | K(G,T) | TGAAATCGAATTCAAAATTTATTTCAGTTCGGTGTGGTTTTTKGGTAAGAAGTAAAAACTGATATAGAACCAAACCATTTGGTTT |
| Zaasp46 | R(G,A) | CTTGACATGAATTCTCTTTCTATTTGTCCCAAAAGAAAAATARAAAAATGTAAGCCAAGGATTTTTTATAAAAATTTTAAACAGG |
| Zaasp48 | R(G,A) | CGAGCATGAGCTCGGAAGTCGAATTCGGGTTGGCCCACCCGARAGCCATGTAATTCATGGTCCCGGTGGCGGCCTCCAATCCGAT |

**Table S2**. SNPs with both sides 42-bp sequences for the russet mapping of sand pear fruit skin.
