# Supplementary material for: An ARF1-binding factor triggering programmed cell death and periderm development in pear russet fruit skin
Source: Hortic Res. 2022 Jan 19;9:uhab061. doi: 10.1093/hr/uhab061 (PMC8947239; doi:10.1093/hr/uhab061)
Supplement: Web_Material_uhab061 [file web_material_uhab061.zip › Table S3.docx]

**Table S3**. SSR and InDel primers selected for the russet mapping of sand pear fruit skin.

| **Name** | **Forward (5' to 3')** | **Reverse (5' to 3')** | **Type** | **Element** |
| --- | --- | --- | --- | --- |
| Zaasp1174 | TAACCTTCGCACGAAGCAAT | TCGAAGTTGACGGAGCTTCT | SSR | TC |
| Zaasp1144 | TTCGGATCACAGCAAGAATG | AATCAAAACCCTCCCCTCAC | SSR | AG |
| Zaasp1145 | TTCGGATCACAGCAAGAATG | AGAGCTCCCTGCTACCTTCC | SSR | AG |
| Zaasp1180 | TGGGTGGCTTTTGTTTCTTT | TTCGGCCTGGAATCAATAAC | SSR | TA |
| Zaasp1186 | AAAATCGACGGTGGAGATTG | AAAACCCTCGCTACACTCCA | SSR | AG |
| Zaasp1195 | GAGTGCCGGCTATCGACTAC | TGGCAGTTACGCAGTTTGAA | SSR | TG |
| Zaasp1196 | TTTTTCCAAGTCTCAAAAATGC | GTGTTGGGATGAACCTGCTT | SSR | TG |
| Zaasp1199 | CGTTGGAACCAATTGTCCTT | TCCTCATCCAAATCCCATGT | SSR | TG |
| Zaasp1204 | CAGCCCGTCTTGGATTTTTA | TCGAGTGTTTTGGTCTTGTCC | SSR | CA |
| Zaasp1147 | GGGGAATTCAGCAGATCAGA | CGCCGGACCGTTTGTAATA | SSR | TC |
| Zaasp1209 | TGCATCAACAAGGAATGCTC | CAGAATTCCGGTGCAAGTTT | SSR | CT |
| Zaasp1211 | ATGATGGAGTCCGAAAATGC | CAAACGAACGAACGATGAGA | SSR | CT |
| Zaasp1213 | TCTGCAATGAGTACCAGCAAA | TGACTGCTGTCATGCTGACTC | SSR | AT |
| Zaasp1226 | GGCGTTTGCCAGAACTGTAT | CCAGCGTTAGGAAGATCGAC | SSR | AG |
| Zaasp1152 | TTCGAACAGAAAGACTGAGGTTT | GGAACTCTTTCCAAAGTTTCTGC | SSR | TG |
| Zaasp800 | AGCATCAAGGAGCTGAGGAA | CAGGTAGCTATCGCAGAGGG | SSR | CT |
| Zaasp810 | CTACCTGCAATCCAAGCCTC | TGCCATATCAGGACACCAGA | SSR | AG |
| Zaasp812 | CTGATTTGAAGGCTTCCACG | AACTTCAAATAACCCCAGCG | SSR | AT |
| Zaasp822 | CTCTTTCATCGACCCTCACA | ACAAGCCCACCAAGTAATCG | SSR | AG |
| Zaasp979 | AACGTGTAGACCAATCTAGC | TGAGCTGACCTGTGGTTTCA | InDel | -ACTAT |
| Zaasp846 | AAACGACGACACTCGAATCC | GCCGAGATTTGCTTCAGAAC | SSR | TC |
| Zaasp791 | CCGGAAGGAGAGAGAAAAGG | TGCACTCCCCATTATGTCAA | SSR | AG |
| Zaasp865 | CACTTGAATCCCCCATTTTC | CGTACGAACAGCCAGTGAGA | SSR | CT |
| Zaasp867 | CCACCTCACCTTATCCCTCA | GCGTTTGCTCCTCGTACTTC | SSR | CT |
| Zaasp868 | AAAAAGTGATGTGTGGATCGC | CTGGTGACTTGCAAGCTAGG | SSR | AT |
| Zaasp873 | CGCTTTCGAACACAAAACAA | CTGGCACTTTGAATTCGCTT | SSR | GA |
| Zaasp1005 | GCAGCTGATCTCCCATTTTC | GCTAACCGTTCGCCTTTATG | SSR | G |
| Zaasp876 | GGCGAACGGTTAGCATAAGA | CTGGTTATGGTCGGTAGCGT | SSR | AG |
| Zaasp1013 | ACAATTCCGCTTACGGACAC | ACAAACTGTTGCTGGTGCAG | SSR | T |
| Zaasp1027 | CAGCCAAACACTCACTGCAT | GTAACCGCGAATTCGAAAAA | SSR | CT |
| Zaasp1029 | TTTCTACACCAGGCATGTCG | AGAAAGAGGGGGCACTTGTT | SSR | ATAC |
| Zaasp792 | GTTTTGCGTCGAAGGTGAAT | AGCTACCGCCGGTTAAGATT | SSR | CGA |
| Zaasp903 | CAGTAGCAATCCCACGGTTT | GCACCGATGCCTGTAACTTT | SSR | AC |
