# Supplementary material for: An ARF1-binding factor triggering programmed cell death and periderm development in pear russet fruit skin
Source: Hortic Res. 2022 Jan 19;9:uhab061. doi: 10.1093/hr/uhab061 (PMC8947239; doi:10.1093/hr/uhab061)
Supplement: Web_Material_uhab061 [file web_material_uhab061.zip › Table S4.docx]

**Table S4.** A list of the genes and their expression in the russet and green fruit skin of sand pear within the scope of the nearest beside markers *Zaasp1147* and *Zaasp1213* of the *PyPPCD1* locus.

| Gene* | **Transcript level (FPKM)** | |  | Gene* | **Transcript level (FPKM)** | |
| --- | --- | --- | --- | --- | --- | --- |
|  | **Russet** | **Green** |  |  | **Russet** | **Green** |
| LOC103927480 | 4.31±1.75 | 7.44±2.01 |  | LOC103927508 | 1.84±0.48 | 3.78±0.54 |
| LOC103927482 | 0.67±0.46 | 0.91±0.37 |  | LOC103927510 | 74.55±12.06 | 67.66±3.89 |
| LOC103927483 | 0.95±0.20 | 1.37±0.40 |  | LOC103927509 | 42.74±4.22 | 36.42±4.37 |
| LOC103927485 | 0.78±0.11 | 0.23±0.19 |  | LOC103927512 | 24.21±1.55 | 9.77±1.17 |
| LOC103927484 | 13.45±1.17 | 19.41±2.19 |  | LOC103927535 | 0.47±0.07 | 0.41±0.39 |
| LOC103927486 | 6.41±0.56 | 10.66±0.58 |  | LOC103927513 | 26.48±2.57 | 25.62±3.34 |
| LOC103927487 | 196.03±17.69 | 237.32±8.36 |  | LOC103927514 | 31.73±4.48 | 27.91±3.09 |
| LOC103927488 | 1.68±0.89 | 1.08±0.77 |  | LOC103927516 | 8.57±2.00 | 6.93±1.19 |
| LOC103927490 | 109.54±14.76 | 54.65±10.97 |  | LOC103927515 | 6.69±0.88 | 3.19±0.50 |
| LOC103927491 | 5.35±1.63 | 9.11±1.83 |  | LOC103927517 | 5.55±0.21 | 17.62±1.28 |
| LOC103927492 | 0.23±0.10 | 0.33±0.17 |  | LOC103927536 | 1.44±0.61 | 0.96±0.14 |
| LOC103927493 | 50.01±4.53 | 50.20±3.95 |  | LOC103949677 | 2.46±0.88 | 1.45±0.77 |
| LOC103927533 | 1.40±0.33 | 1.41±0.35 |  | LOC103949681 | 21.75±2.70 | 21.87±2.53 |
| LOC108865333 | 0.77±0.24 | 0.88±0.33 |  | LOC103949680 | 283.07±90.35 | 259.69±81.67 |
| LOC103927495 | 41.29±2.77 | 37.94±3.96 |  | LOC103949678 | 125.32±23.70 | 119.60±22.08 |
| LOC103927497 | 27.20±3.23 | 21.28±2.36 |  | LOC103949682 | 4.46±0.17 | 4.62±0.74 |
| LOC103927496 | 16.30±1.30 | 15.46±1.98 |  | LOC103949683 | 17.29±2.34 | 17.93±3.25 |
| LOC103927498 | 14.01±12.87 | 30.14±14.21 |  | LOC103949685 | 36.87±7.92 | 31.50±7.49 |
| LOC103927504 | 124.89±66.65 | 198.00±124.35 |  | LOC103949689 | 18.49±9.48 | 5.48±3.68 |
| LOC108865400 | 0.23±0.08 | 1.48±0.57 |  | LOC103949688 | 2.74±1.11 | 2.52±1.06 |
| LOC103927503 | 154.70±67.46 | 222.88±118.51 |  | LOC103949686 | 30.91±16.49 | 10.09±6.57 |
| LOC103927502 | 80.23±26.01 | 190.07±156.75 |  | LOC103949691 | 0.11±0.17 | 0.18±0.09 |
| LOC103927501 | 0.75±0.26 | 1.84±0.23 |  | LOC103949693 | 0.23±0.18 | 0.03±0.03 |
| LOC103927507 | 5.33±0.72 | 4.54±0.74 |  | LOC103949697 | 1.61±0.25 | 0.60±0.11 |

*The gene marked in grey has low expression in both russet and green fruit skin of sand pear.
