# Supplementary material for: An ARF1-binding factor triggering programmed cell death and periderm development in pear russet fruit skin
Source: Hortic Res. 2022 Jan 19;9:uhab061. doi: 10.1093/hr/uhab061 (PMC8947239; doi:10.1093/hr/uhab061)
Supplement: Web_Material_uhab061 [file web_material_uhab061.zip › Table S5.docx]

**Table S5.** Repeats of the GAC element in *LOC103949685* alleles in 49 pear cultivars.

| **Cultivar** | **Fruit skin color** | **SSR type*** |
| --- | --- | --- |
| Sazansuido, Jinqiu, Natsusiziku, Akizuki, Tsukuba 49 | **Green** | j k |
| Tsukuba 45 | **Green** | i k |
| Azumanishiki, Cuilv, Chikusui | **Green** | j j |
| Hangqing, Xinya, Whangkeumbae, Kosui, Cuiguan, Liuyuesu, Xizilv, Mantianhong, Cuiyu, Hongtaiyang, Dayali, Longquansu, Hakataao | **Green** | i j |
| Zhenzhuli, Chuxialv, Xueqing, Xinlvshui, Fengxiang, Osa-nijisseiki, Nikko, Meirensu, Sucui No.1 | **Green** | i i |
| Yiwusanhuali | **Russet** | h h |
| Syusui, Zaoshuhuangpi, Housui, Akibae, Zhenxiang, Qingxiang-BM, Qingxiang, Yanhuang | **Russet** | h i |
| Kisui, Xinyu, Mixue, Nansui, Ryouhou, Daguohuanghua, Yuguan, Wakahikari, Yiwumeili | **Russet** | h j |

*h-k, repeats (n) of the GAC element in the SSRs (GAC)_n_GAT is 5, 6, 7, 8, respectively.
