# Supplementary material for: An ARF1-binding factor triggering programmed cell death and periderm development in pear russet fruit skin
Source: Hortic Res. 2022 Jan 19;9:uhab061. doi: 10.1093/hr/uhab061 (PMC8947239; doi:10.1093/hr/uhab061)
Supplement: Web_Material_uhab061 [file web_material_uhab061.zip › Table S6.docx]

**Table S6.** The top 10 stuctural analogs of PyPPCD1 identified in PDB using I-TASSER platform.

| Rank | PDB Hit | TM-score^a^ | RMSD^b^ | IDEN^c^ | Cov^d^ | Classification | Organism(s) |
| --- | --- | --- | --- | --- | --- | --- | --- |
| 1 | 5cdcB | 0.746 | 3.69 | 0.053 | 0.928 | VIRUS | Israeli acute paralysis virus |
| 2 | 1b35C | 0.636 | 3.7 | 0.077 | 0.795 | VIRUS | Cricket paralysis virus |
| 3 | 3napC | 0.629 | 3.94 | 0.073 | 0.808 | VIRUS | Triatoma virus |
| 4 | 5g52C | 0.613 | 4.24 | 0.064 | 0.798 | VIRUS | Deformed wing virus |
| 5 | 5j96C | 0.606 | 4.12 | 0.075 | 0.785 | VIRUS | Slow bee paralysis virus |
| 6 | 6iicC | 0.596 | 3.82 | 0.052 | 0.756 | VIRUS | Mud crab virus |
| 7 | 5mqcC | 0.594 | 3.98 | 0.065 | 0.75 | VIRUS | Black queen cell virus |
| 8 | 4qpgC | 0.574 | 4.1 | 0.077 | 0.746 | VIRUS | Human hepatitis A virus |
| 9 | 5oypC | 0.573 | 4.25 | 0.082 | 0.752 | VIRUS | Sacbrood virus of honeybee |
| 10 | 1mec3 | 0.534 | 4.06 | 0.065 | 0.697 | VIRUS | Mengo virus |

TM-score^a^ is a measure of global structural similarity between query and template protein. RMSD^b^, root-mean-square deviation, here is the RMSD between residues that are structurally aligned by TM-align.

IDEN^c^ is the percentage sequence identity in the structurally aligned region.

Cov^d^ represents the coverage of the alignment by TM-align and is equal to the number of structurally aligned residues divided by length of the query protein.
