# Supplementary figures and images for: An ARF1-binding factor triggering programmed cell death and periderm development in pear russet fruit skin
Source: Hortic Res. 2022 Jan 19;9:uhab061. doi: 10.1093/hr/uhab061 (PMC8947239; doi:10.1093/hr/uhab061)

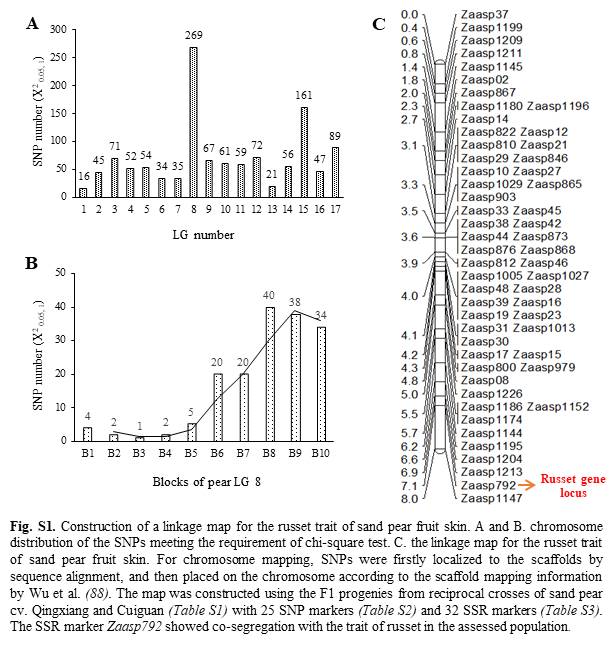

Supplement: Web_Material_uhab061 [file web_material_uhab061.zip › Fig. S1.jpg]

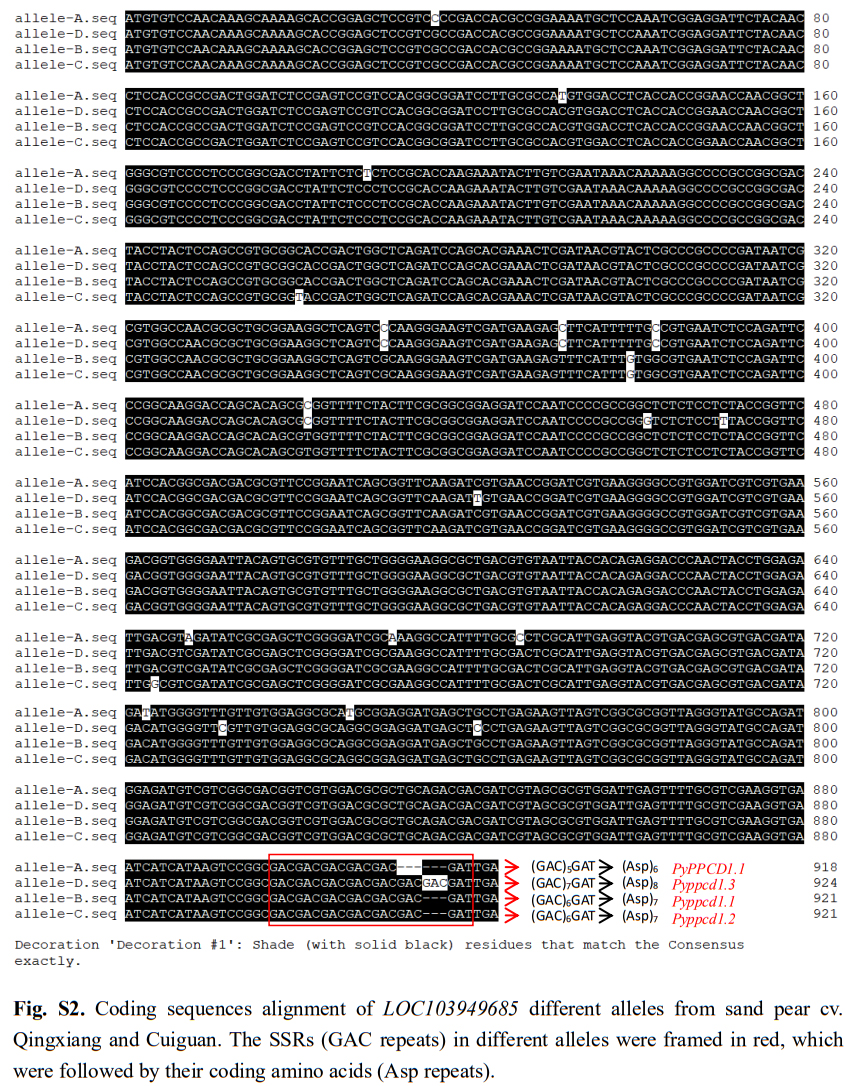

Supplement: Web_Material_uhab061 [file web_material_uhab061.zip › Fig. S2.jpg]

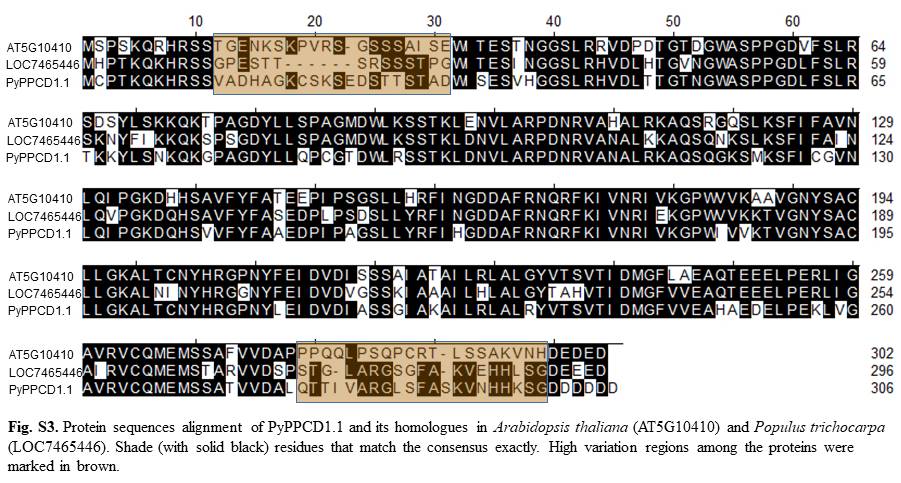

Supplement: Web_Material_uhab061 [file web_material_uhab061.zip › Fig. S3.jpg]

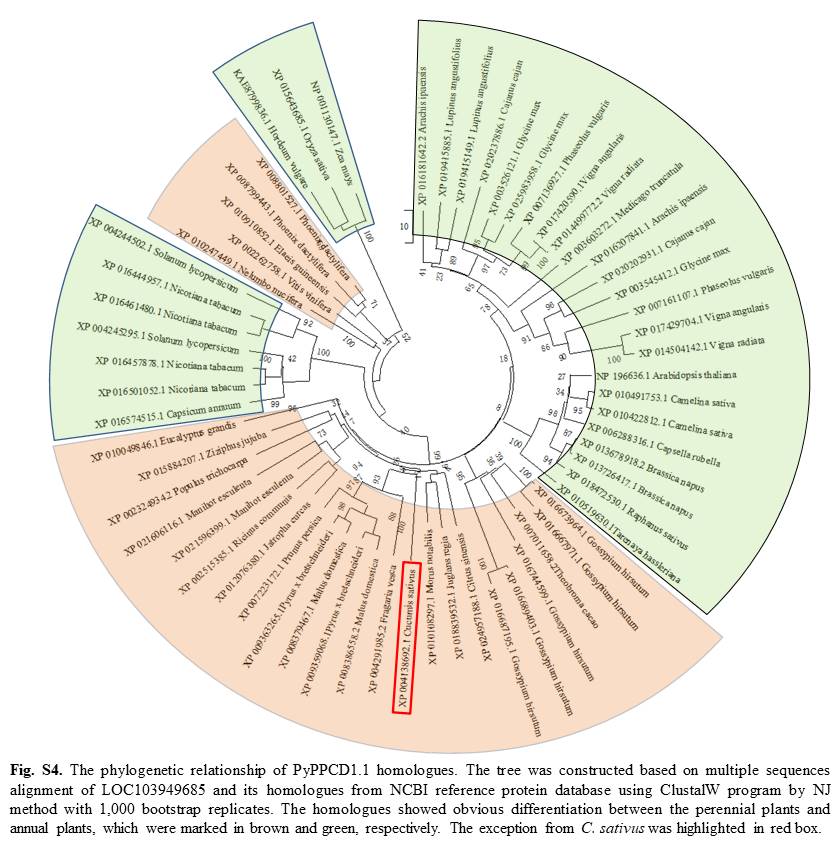

Supplement: Web_Material_uhab061 [file web_material_uhab061.zip › Fig. S4.jpg]

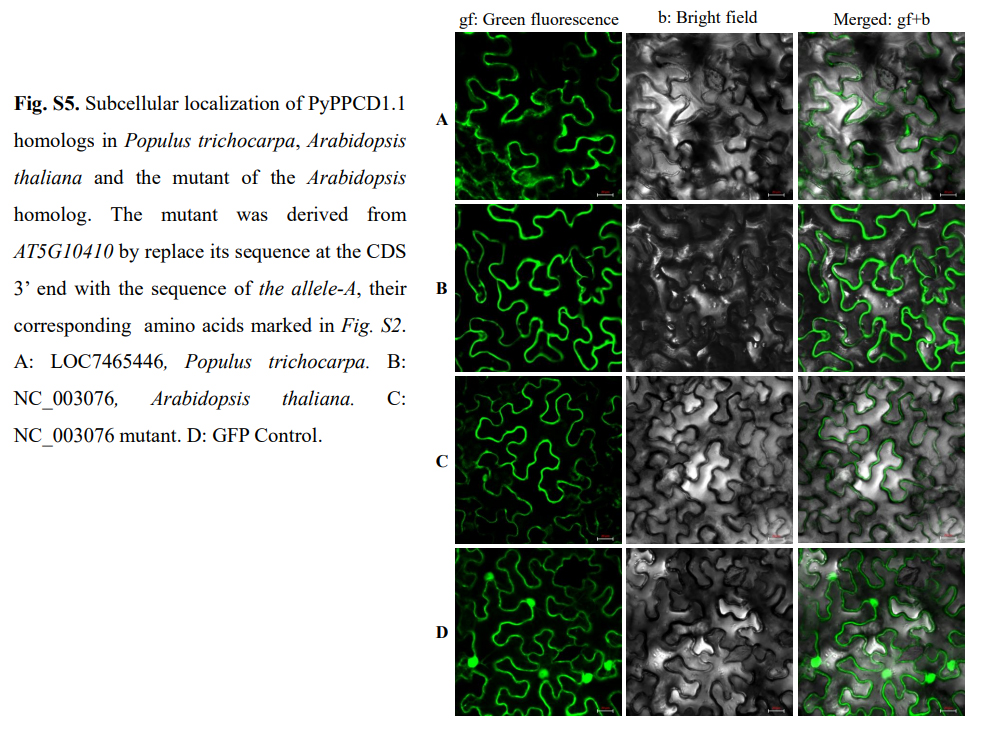

Supplement: Web_Material_uhab061 [file web_material_uhab061.zip › Fig. S5.jpg]

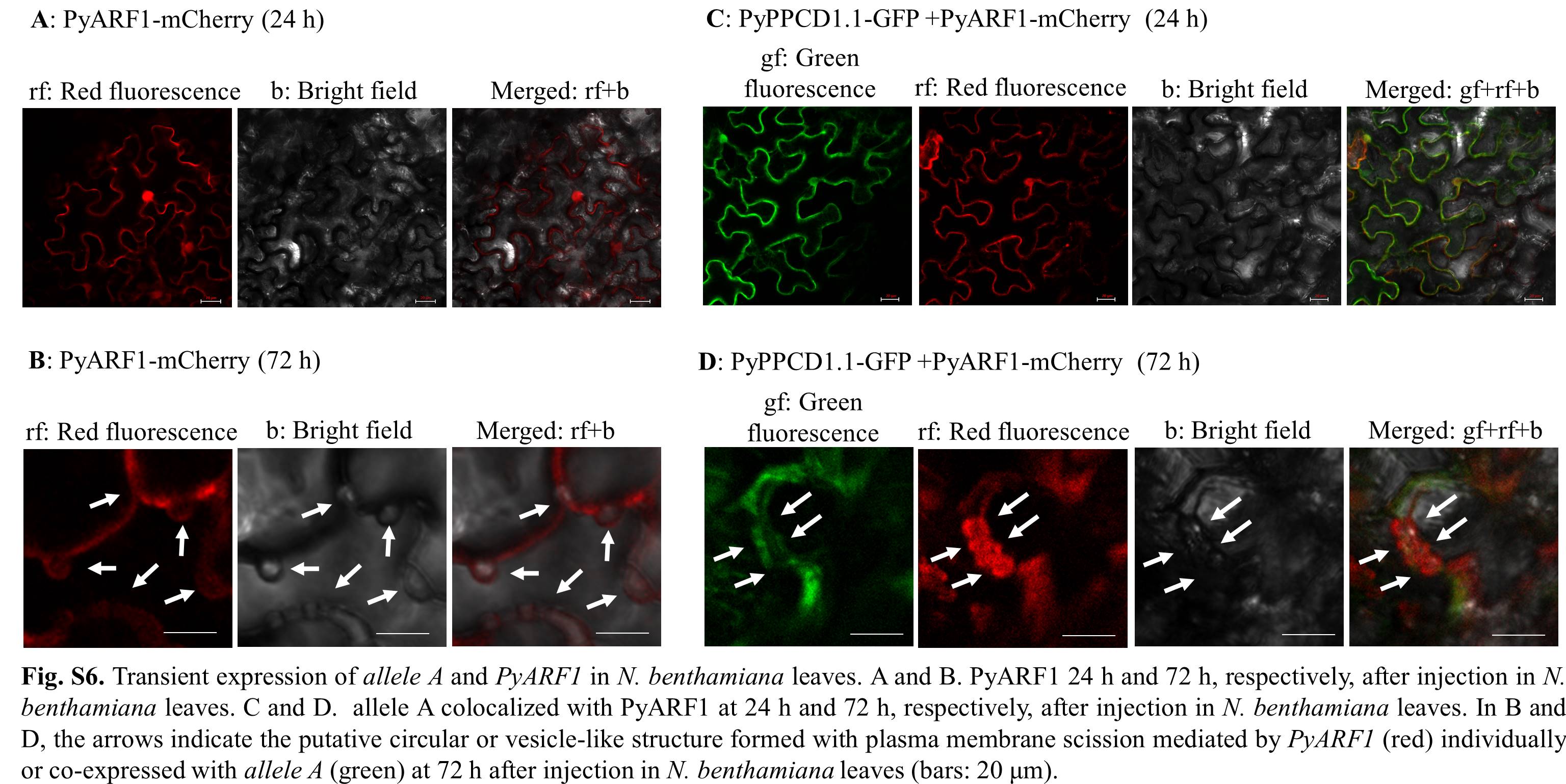

Supplement: Web_Material_uhab061 [file web_material_uhab061.zip › Fig. S6.jpg]

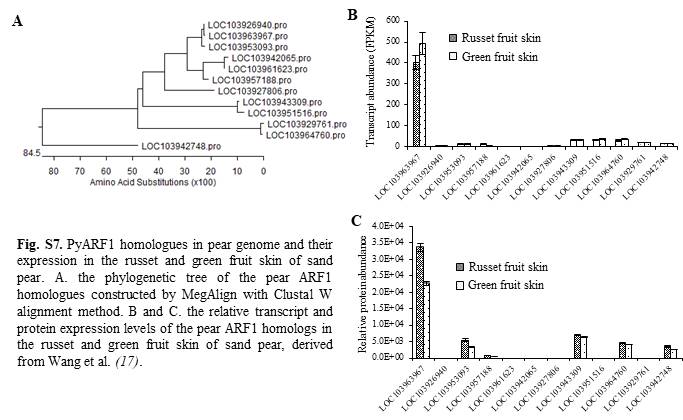

Supplement: Web_Material_uhab061 [file web_material_uhab061.zip › Fig. S7.jpg]
