# Supplementary material for: An ARF1-binding factor triggering programmed cell death and periderm development in pear russet fruit skin
Source: Hortic Res. 2022 Jan 19;9:uhab061. doi: 10.1093/hr/uhab061 (PMC8947239; doi:10.1093/hr/uhab061)
Supplement: Web_Material_uhab061 [file Fig. S2.pdf › Fig. S2.pdf]

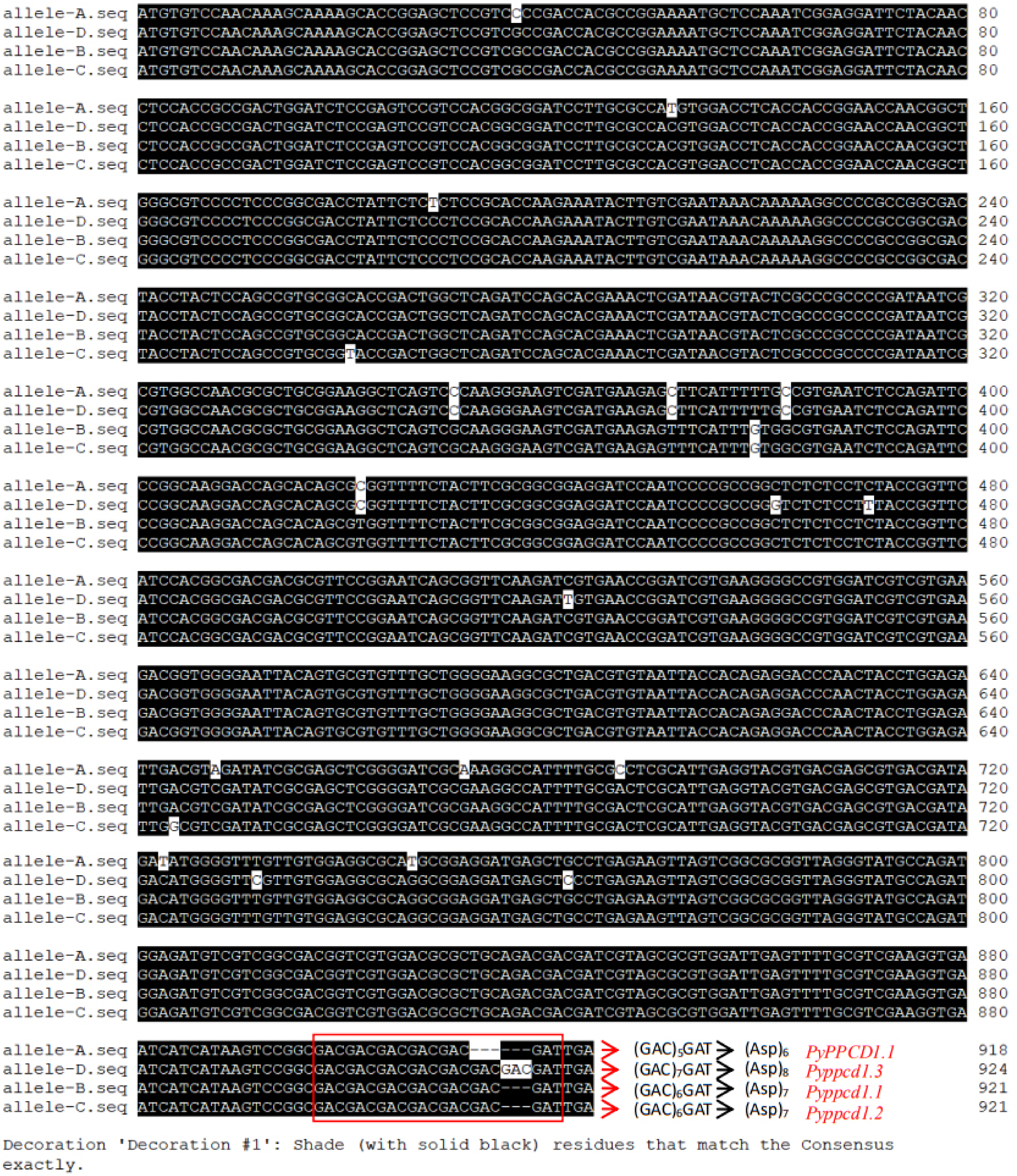

Fig. S2. Coding sequences alignment of *LOC103949685* different alleles from sand pear cv. Qingxiang and Cuiguan. The SSRs (GAC repeats) in different alleles were framed in red, which were followed by their coding amino acids (Asp repeats).
